# Mesoporous Magnetic Cysteine Functionalized Chitosan Nanocomposite for Selective Uranyl Ions Sorption: Experimental, Structural Characterization, and Mechanistic Studies

**DOI:** 10.3390/polym14132568

**Published:** 2022-06-24

**Authors:** Ahmed A. Al-Ghamdi, Ahmed A. Galhoum, Ahmed Alshahrie, Yusuf A. Al-Turki, Amal M. Al-Amri, S. Wageh

**Affiliations:** 1 Department of Physics, Faculty of Science, King Abdulaziz University, Jeddah 21589, Saudi Arabia; agamdi@kau.edu.sa (A.A.A.-G.); aalshahri@kau.edu.sa (A.A.); 2 Nuclear Materials Authority, El-Maadi, Cairo P.O. Box 530, Egypt; galhoum_nma@yahoo.com; 3 Centre of Nanotechnology, King Abdul-Aziz University, Jeddah 21589, Saudi Arabia; 4 Department of Electrical and Computer Engineering, Faculty of Engineering, King Abdulaziz University, Jeddah 21589, Saudi Arabia; yaturki@yahoo.com; 5 K. A. CARE Energy Research and Innovation Center, King Abdulaziz University, Jeddah 21589, Saudi Arabia; 6 Physics Department, Rabigh College of Science and Arts, King Abdulaziz University, P.O. Box 344, Rabigh 21911, Saudi Arabia; amsalamri@kau.edu.sa

**Keywords:** mesoporous magnetic chitosan nanocomposite, cysteine, uranyl ions, mechanistic studies, XPS, uptake kinetics and sorption isotherms

## Abstract

Nuclear power facilities are being expanded to satisfy expanding worldwide energy demand. Thus, uranium recovery from secondary resources has become a hot topic in terms of environmental protection and nuclear fuel conservation. Herein, a mesoporous biosorbent of a hybrid magnetic–chitosan nanocomposite functionalized with cysteine (Cys) was synthesized via subsequent heterogeneous nucleation for selectively enhanced uranyl ion (UO_2_^2+^) sorption. Various analytical tools were used to confirm the mesoporous nanocomposite structural characteristics and confirm the synthetic route. The characteristics of the synthesized nanocomposite were as follows: superparamagnetic with saturation magnetization (*M*_S_: 25.81 emu/g), a specific surface area (S_BET_: 42.56 m^2^/g) with a unipore mesoporous structure, an amine content of ~2.43 mmol N/g, and a density of ~17.19/nm^2^. The experimental results showed that the sorption was highly efficient: for the isotherm fitted by the Langmuir equation, the maximum capacity was about 0.575 mmol U/g at pH range 3.5–5.0, and Temperature (25 ± 1 °C); further, there was excellent selectivity for UO_2_^2+^, likely due to the chemical valent difference. The sorption process was fast (~50 min), simulated with the pseudo-second-order equation, and the sorption half-time (t_1/2_) was 3.86 min. The sophisticated spectroscopic studies (FTIR and XPS) revealed that the sorption mechanism was linked to complexation and ion exchange by interaction with S/N/O multiple functional groups. The sorption was exothermic, spontaneous, and governed by entropy change. Desorption and regeneration were carried out using an acidified urea solution (0.25 M) that was recycled for a minimum of six cycles, resulting in a sorption and desorption efficiency of over 91%. The as-synthesized nanocomposite’s high stability, durability, and chemical resistivity were confirmed over multiple cycles using FTIR and leachability. Finally, the sorbent was efficiently tested for selective uranium sorption from multicomponent acidic simulated nuclear solution. Owing to such excellent performance, the Cys nanocomposite is greatly promising in the uranium recovery field.

## 1. Introduction

Uranium is the most significant element for nuclear energy production, but it is a limited resource, and a shortage is projected in the near future. It is also highly toxic. The extraction of uranium and the development of nuclear power can produce hazardous and radioactive waste [1,2,3,4,5]. Consequently, uranium separation and recovery are important processes in terms of both uranium resource exploitation and environmental preservation. Sorption (sometimes called solid-phase extraction) is a promising method for recovering metal ions from dilute effluents, due to its low environmental impact, quick kinetics, excellent selectivity, ease of handling, and great efficiency [2,6,7,8,9]. Recent progress in uranium(VI) ion sorption from contaminated water has been conducted using various sorbents such as organic materials, e.g., cellulose/chitosan-based sorbent materials [1,2,3,4,5], functionalized synthetic polymers [1,10], hydrogel [11], and aerogel [12]. Moreover, porous materials have been widely studied in the field of uranium extraction from seawater [1,2,3]. Clays, metal oxides, and AL-based LDH are examples of inorganic materials [13]. Magnetic nanocomposites, as well as nanomaterials, are examples of composite and metal–organic frameworks (MOFs) [2,14].

As an alternative to traditional synthetic resins, biosorption and biopolymers such as chitosan and cellulose have recently attracted a lot of attention [1,2,3,4,5]. For water remediation, chitosan-based polymers have been widely investigated [15,16,17]. Chitosan (CT), a reactive biopolymer (amino-polysaccharide obtained by alkaline acetylation of chitin), has received great attention because it is a renewable resource, abundant, and biodegradable [1,2,3,4,5]. CT is a good sorbent for metal ions, and bears hydroxyl and amine reactive functionalities that can be bound in a variety of mechanisms: metal anions are attracted to each other through ion exchange/electrostatic attraction, and metal cations are chelated [1,2,3,4,5], and open up a world of further functionalization. Chitosan appears to be a good starting material for chelating resin production [8,18]. Resistance to mass transfer is frequently the key limiting phase in sorption processes. The difficulty is to increase porosity or use small-size materials at the expense of more complicated solid/liquid separation at the sorption equilibrium [10,19].

Magnetic-based composites can overcome this disadvantage. Nanocomposites are multiphase materials made up of nanometric iron oxide (Fe_3_O_4_) particles incorporated in a polymer matrix [17]. This is the approach taken in the current study to develop a new sorbent for uranium recovery [1,2,3,4,5]. The art of building innovative multiphase nanomaterials for efficient uranium sorption involves the fabrication of an organic–inorganic functional hybrid nanocomposite (high capacity and selectivity). Furthermore, an external magnetic field can be used to recover and collect the sorbents [5,20]. This is the main reason for the manufacture of magnetic sorbent synthesis based on chitosan. Grafting diverse reactive functional groups enhances sorption capacity, target metal selectivity, and the pH range for efficient sorption [1,19]. Several chelating ligands can be used for the sorption of uranium ions, such as catechol, iminodiacetic acid [1,2,3,4,5], aminophophaonate (acids and esters) [3,8], polyamines [21], magnetic Schiff’s base chitosan composite [21], phenylarsonic acid and amino acid moieties functionalized crosslinked chitosan [1,2,3,4,5], and tryptophan-porous organic polymer [22]. Amino acids are chemical organic compounds rich with amine (–NH_2_) and carboxylic acid (–COOH) (i.e., considered as hard bases), as well as a side chain (e.g., –OH, –SH), and have a strong affinity for uranium (hard acid), according to Persson’s definition of the HSAB hypothesis [1,2,3,4,5].

To date, no articles have reported the extensive and deep characterization needed for mechanistic studies of the synthesis procedures, chemical modification, sorption, and desorption mechanism for uranyl ions. Therefore, it is highly desirable to develop an extensive discussion of these points. Thus, herein, a co-precipitation of a magnetic nanoparticle coating with a thin film layer of chitosan was conducted, resulting in a magnetic chitosan nanocomposite, which was followed by further modification with cysteine via a continuous reaction. The physicochemical characteristics were characterized by elements analysis, XRD, pHpzc, TEM, VSM, FTIR, and XPS. The magnetic chitosan nanocomposite formation and cysteine grafting mechanism are discussed in detail. Experimental results and spectroscopic (FTIR and XPS) studies were used to predict the uranium surface complexation models. Sorption of UO_2_^2+^ onto the Cys nanocomposite as functions of sorbent dose, pH, contact time, uptake kinetics, sorption isotherms, thermodynamic characterization, and selectivity was analyzed. Further, the sorbent regeneration and re-usability were examined. Finally, mechanistic studies of the nanocomposite formation (magnetite preparation and chemical modification stages), sorption, and desorption were studied and are discussed in detail.

## 2. Materials and Methods

### 2.1. Materials

Chitosan (DD: 93%) was supplied by Acros Organics. Epichlorohydrin (CAS:106-89-8), 1,4-dioxane (CAS: 123-91-1), Cysteine (CAS: 52-90-4), Arsenazo III (A.R. grade), FeCl_2_.4H_2_O (≥99%), and FeCl_3_ (≥99.99%) were provided from Sigma-Aldrich. All reagents were used exactly as they were given without any purification. Uranyl acetate (UO_2_(OCOCH_3_)_2_·2H_2_O) (Sisco Research Laboratories Pvt. Ltd., Mumbai, India) was dissolved in ultrapure water to prepare a stock solution (1000 mg U/L). Uranium content was analyzed using a UV/Vis spectrophotometer [23].

### 2.2. Sorbent Synthesis

#### 2.2.1. Chitosan–Magnetite Nanocomposite Preparation

A hybrid magnetic chitosan nanocomposite was created by hydrothermally co-precipitating Fe^(II)^ and Fe^(III)^ mixture with NaOH in the presence of chitosan in a one-step process [4,5]. Chitosan powder (2 g) was steeped and dissolved in acetic acid solution (200 mL, 5% *w*/*w*) with ferrous chloride (FeCl_2_, 2.42 g) and ferric chloride (FeCl_3_, 3.82 g) (1:2 molar ratio, respectively). At 40–45 °C, the mixture solution was precipitated by adding NaOH (2 M) dropwise with steady stirring at pH 10.5–11. The suspension was heated for 1 h at 90 °C while stirring constantly, and the particles were magnetically collected. The freshly magnetic chitosan nanocomposite was chemically enhanced by crosslinking with an epichlorohydrin solution (0.01 M) at 1:1 mass ratio under alkaline conditions (pH ~ 10) [6]. The suspension mixture was heated at 50 °C for 2 h with constant stirring. At the end, the cross-linked magnetic chitosan nanocomposite was magnetically collected and washed extensively with ethanol/water to eliminate any unreacted reagent.

#### 2.2.2. Nanocomposite Functionalization

**Scheme 1:**
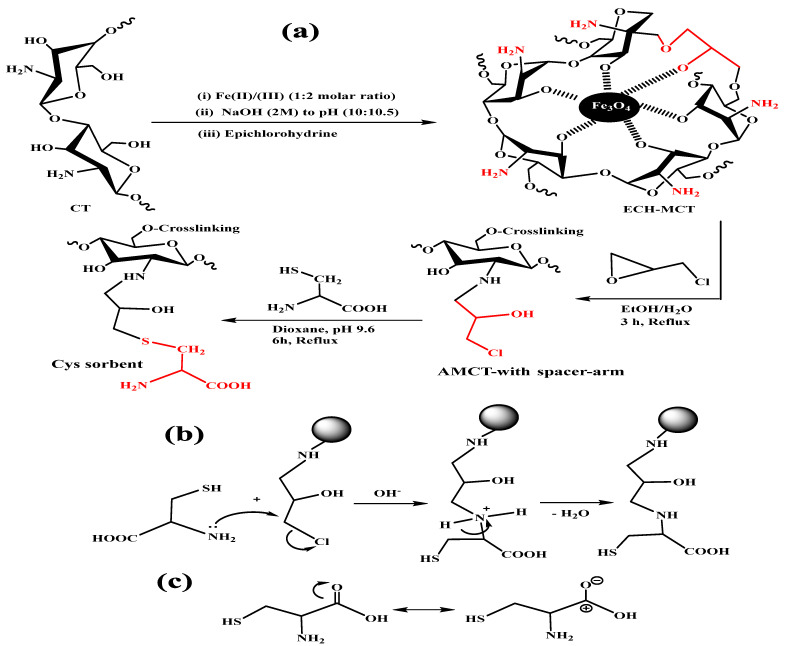
Synthesis of Cysteine−modified magnetic chitosan nanocomposite (**a**), and revised process for cysteine grafting via SN2 mechanism (**b**,**c**).

Cysteine was functionalized with cross-linked magnetic chitosan nanocomposite as depicted in Scheme 1 using a generic approach. The cross-linked magnetic chitosan nanocomposite was suspended in aqueous ethanol solution (75 mL, 1:1 in *v*/*v*) before 10 mL of epichlorohydrin was added; refluxing the mixture at 65–70 °C for 3 h yielded the desired results [7]. The product was filtered and rinsed thoroughly with ethanol and ultrapure water. Finally, the activated product and cysteine (8 g) were suspended in 100 mL of dioxane, and the mixture pH was adjusted to 9.5–10. The mixture was refluxed at 95 °C for 8 h. The final product was magnetically separated and washed extensively with ethanol and ultrapure water. The sorbent was finally freeze-dried.

### 2.3. Characterization

(C, H, N, and S) was determined using an automatic analyzer (CHNS Vario EL III−elementar analyzer, Elementar, Langenselbold, Germany). A Philips X−ray generator type PW 3710/31, Cu Kα (λ = 1.544) (Philips model, Eindhoven, The Netherlands) was used to measure X−ray diffraction. The FTIR spectra were analyzed by a Thermo-Fisher Nicolet IS10 spectrometer (Waltham, MA, USA). The morphology and size distribution were analyzed by TEM-JEOL-2100 transmission electron microscope (JOEL, Tokyo, Japan). The magnetic characteristics were determined using a vibrating sample magnetometer (VSM; 730T, Lakeshoper Cryotonics, inc., Westerville, OH, USA). A Quantachrome NOVA 3200 (Boynton Beach, FL, USA) analyzer was used to measure the surface area using N_2_-adsorption isotherms under a degassing temperature of 40 °C for 12 h, while the pore volume was measured according to the BJH method. The K-Alpha spectrometer (Thermo Fisher Scientific, Waltham, MA, USA) was used to acquire X−ray photoelectron spectroscopy (XPS) data using a monochromatic Al Kα-radiation source (200 W). The sorbent’s pH point of zero charge (pH_PZC_) was measured using the pH−drift method [7,8], and the sorbent was equilibrated for 24 h under agitation with a series of 0.1 M NaCl solutions with different initial pH-values (pH_0_); the equilibrium pH (pH_eq_) was recorded, and the pH_PZC_ was the pH at which ΔpH = 0.

### 2.4. Batch Sorption and Desorption Experiments

The sorption properties of synthesized sorbent were investigated. A constant UO_2_^2+^ solution volume (20 mL) with a definite concentration (C_0_, mmol U/L) was agitated and equilibrated with a given amount of sorbent (10 mg) for 180 min at room temperature (298 ± 1 K), 200 rpm and initial pH (pH_0_ 4.0), except when the following were present: the effect of initial pH (the pH_0_: 1.3–5.5), equilibration time (from 0 min to 150 min. at pH_0_: 4.0), isotherm at various concentrations (C_0_: 0.21–1.26 mmol U/L), temperature (at 298–328 ± 1 K), and sorbent dose (SD: 0.25–1.5 g/L) on the sorption capacities. After equilibrium and phase separation, the initial, residual UO_2_^2+^ concentration at equilibrium and at different interval times (C_0_, C_eq_, and C_t_: mmol U/L) were determined by the Arsenazo III method (using a double beam UV-11601, UV-Vis spectrophotometer (Shimadzu Corporation, Kyoto, Japan)). The sorption capacity (q_eq_, mmol U/g) was evaluated by the mass balance equation and the distribution coefficient (D: q_eq_/C_eq_) in L/g, as shown in Equations (1) and (2), respectively:

(1) $$q_{\mathrm{eq}}=\frac{(C_{o}-C_{\mathrm{eq}})\times \;V}{m}$$

(2) $$D\;=\frac{\left(C_{o}-C_{\mathrm{eq}}\right)}{C_{o}}\times \frac{V}{m}\;$$

where m (g) is the amount of sorbent, and V (L) is the solution’s volume. Experimental conditions are stated in the figure captions: in most cases, the equilibrium time was 50 min (at room temperature, i.e., 298 K), and the pH_0_ was set at 4.0. All values are means of duplicated with standard deviation ±4–5%.

The influence of competing cations on the uranyl sorption in a synthetic simulated nuclear industry effluent was studied in order to explore the selectivity toward U(VI). A standardized investigation was conducted utilizing some of the most common metals present in nuclear industry effluents, such as REs(III): La, Ce, Eu, and Yb, and other co-ions (Co, Cd, Cs, Sr, and Ba). Equimolar multi-solutions (as the saline content of nuclear industry wastewater is usually considerable) were made for this investigation using their nitrate and chloride salts. The sorption studies were conducted at room temperature (298 ± 1 K), and with a 0.5 g/L sorbent dose for 60 min and agitation (200 rpm). After filtration, the remaining concentrations were determined by atomic absorption spectrometry (AAS: a Solar Unicam 969, Thermo Scientific, USA) for Co, Cd, Cs, Sr, and Ba, and uranium and rare earth ions were determined by ICP spectrometry (ICP-OES, Prism ICP, Teledyne Leeman Labs., Hundson, NH, USA).

The optimization of the regeneration/recycling (for six successive sorption/desorption cycles) of the sorbent was conducted using acidified urea (10 mL, 0.5 M) with a fixed sorbent weight (10 mg). The contact time was set to 30 min at 200 rpm and at 298 ± 1 K. At each phase, the sorption and desorption efficiency for six cycles were evaluated and compared to the value reached for the first cycle. The equations that describe desorption efficiency (D_E_), and regeneration efficiency (RE) are Equations (3) and (4), respectively:

(3) $$D_{E}=\frac{C_{D}\times {\;V}_{L}\times 100}{m_{d\;}\times {\;q}_{d}}$$

(4) $$\mathrm{RE}\;=\frac{q_{d}\times 100}{q_{e}}$$

where q_d_ (mmol U/g) is the sorption capacity before first desorption, C_D_ (mmol U/L) is the U concentration in the eluate, V_L_ is the volume of the eluent, q_e_ (mmol U/g) is the sorption capacity at the first cycle and m_d_ (g) is the amount of sorbent employed in the desorption stage.

## 3. Results and Discussion

### 3.1. Basics of Hybrid Nanocomposite

A magnetic chitosan nanocomposite was synthesized by heterogeneous nucleation via a hydrothermal one-step co-precipitation of Fe^(II)^ and Fe^(III)^ ions using NaOH in the presence of chitosan at pH ~ 10.5. First, Fe_3_O_4_ nanocrystals were used for nucleation of the chitosan film layer from the solution [4]. The simple overall reaction for synthesizing nano-magnetic (Fe_3_O_4_) nanoparticles under alkaline conditions was conducted using different bases, BOH (B^+^: NH_4_^+^/Na^+^/K^+^). The reaction of Fe^(II)/(III)^ precursor solution and the precipitant base (BOH) was slightly exothermic. The iron oxide (ferrous–ferric oxide (FeO/Fe_2_O_3_)) particle formation started as soon as NaOH was added into the iron salts. The hydrolysis products Fe(OH)^+^ and Fe(OH)_2_^+^ interacted to form ferrihydrite, which preferentially precipitated (Fe(OH)_2(s)_ ↔ Fe(OH)^+^_(aq)_ + OH^−^) and turned into magnetite (Fe_3_O_4_), which was formed as a result of thermodynamic instability [9], as the following mechanism shows (Equations (5)–(11)) [9,21].

Deprotonation step:

(5) $${\mathrm{Fe}}^{3+}+xH_{2}O\;\to 2\mathrm{Fe}{\left(\mathrm{OH}\right)}_{x}^{3-x}+xH^{+}\;$$

(6) $${\mathrm{Fe}}^{2+}+yH_{2}O\;\to \mathrm{Fe}{\left(\mathrm{OH}\right)}_{x}^{2-x}+yH^{+}$$

Ferrihydrite intermediate production:

(7) $$2\mathrm{Fe}{\left(\mathrm{OH}\right)}_{2}^{+}+\mathrm{Fe}{\left(\mathrm{OH}\right)}^{+}+3{\mathrm{OH}}^{-}\to {\left({\mathrm{Fe}}^{3+}\right)}_{2}\left({\mathrm{Fe}}^{2+}\right){\left({\mathrm{OH}}^{-}\right)}_{8}$$

Oxidation and dehydration step:

(8) $$2\mathrm{Fe}{\left(\mathrm{OH}\right)}_{x}^{3-x}+\mathrm{Fe}{\left(\mathrm{OH}\right)}_{y}^{2-y}\;\to {\mathrm{Fe}}_{3}O_{4}+\frac{2x+y}{2}H_{2}O\;$$

Magnetite is formed when ferrihydrite dehydrates:

(9) $${\left({\mathrm{Fe}}^{3+}\right)}_{2}\left({\mathrm{Fe}}^{2+}\right){\left({\mathrm{OH}}^{-}\right)}_{8}\to {\mathrm{Fe}}_{3}O_{4}+4H_{2}O\;$$

Overall reaction:

(10) $$2{\mathrm{Fe}}^{3+}+{\mathrm{Fe}}^{2+}+8{\mathrm{OH}}^{-}\;\to {\mathrm{Fe}}_{3}O_{4}+4H_{2}O\;$$

(11) $$2{\mathrm{FeCl}}_{3}+{\mathrm{FeCl}}_{2}+8\mathrm{BOH}\;\to {\mathrm{Fe}}_{3}O_{4}+8\mathrm{BCl}\;+4H_{2}O$$

This reaction took place under N_2(g)_, to prevent Fe^(II)^ from oxidizing to Fe^(III)^. The magnetite can be oxidized to other ferric hydroxide phases, e.g., maghemite by the ion transfer process (Fe_3_O_4_ + 2H^+^ → *γ*-Fe_2_O_3_ + H_2_O + Fe^2+^) [21], goethite (α-FeOOH), and sometimes hematite (4Fe_3_O_4_ + 4O_2_ → 6Fe_2_O_3_) [21,24]. To avoid both dissolving in acidic environments, magnetite–chitosan nanocomposites are cross-linked using epichlorohydrin, and sorption capacity loss is prevented when amine groups are included (i.e., aldehyde crosslinking is used) [7]. Further, the crosslinking unifunctional agent is employed to generate covalent connections with the C-atoms attached to chitosan’s hydroxyl groups (related to the epoxide ring rupture and a chlorine atom being released) [5].

The synthesis of the magnetic-cysteine-functionalized chitosan nanocomposite is depicted in Scheme 1a. Finally, in alkaline dioxane solution and heating, a typical substitution nucleophilic bimolecular (SN2) reaction occurs between nucleophilic sites on the cysteine moiety (in terminal –NH_2_ and/or –SH) and the carbon linked with the Cl element, and is considered to be responsible for the chemical reaction/modification. The alkaline aprotic solvent promotes this reaction (Scheme 1b). Carboxylic groups are electron withdrawing sites that help to remove the lone electron pair on the N-atom from cysteine, making them less nucleophilic (Scheme 1c): this might explain possibility of –SH groups are preferred over –NH_2_. However, the strength of –NH stretching vibration increased significantly in the FTIR spectra, but free C–S stretching vibration was observed at 742 cm^−1^ [10]. Although several amino groups of the amino acid may be implicated in immobilization, it appears that the majority of grafting occurs via the alternative substituent via –SH sites.

### 3.2. Sorbent Characterization

#### 3.2.1. Element Analysis

The elemental analysis (CHNS) of the materials (in *w*/*w*) was compared during the synthesis procedure (Table 1). When raw chitosan’s (r-CT) and magnetic chitosan’s (MCT) organic fractions (related to C and N elements) were compared, the weight percentage of raw chitosan was found to be significantly lower (~52.7–53.9%): this is related to the presence of iron oxide (Fe_3_O_4_) nanoparticles. The organic fraction was ~46.5%, whereas magnetite accounted for ~53.5% of the total weight, which was consistent with the mass balance experiments. Surprisingly, the increase in the mass of the magnetite chitosan nanocomposite was about 5.35 g compared to the theoretical calculation (4.0 g), based on the initial chitosan to magnetite weight ratio (2 g:2 g). After the crosslinking step, the N-content (mmol%) of cross-linked magnetic chitosan (CMCT) decreased from 2.56 to 2.17, due to increasing epichlorohydrin binding (N-atoms; thus, N-content decreased in the final products), which was consistent with the slight reductions in the C and H contents. Meanwhile, after the activation and insertion of the terminal –Cl atom via epichlorohydrin reaction, the molar mass of activated magnetic cross-linked chitosan (AMCT) steadily increased; therefore, the C, H, N contents were “diluted”, and the changes in the weight percent (Δwt., %) were 10.8, 14.4, and 18.3%, respectively. By analogy, a similar result was observed from immobilizing Cl atoms by the reaction of thionyl chloride (SOCl_2_) for the subsequent immobilization of amine-bearing moieties. After chlorination, the fraction of C and H elements strongly deceased by about 21.8% and 24.5%, respectively, as a result of the Cl atom influence on the final product [12]. Finally, after cysteine grafting, the S content (wt., %) of the obtained Cys nanocomposite (Cys) reached 3.01% (0.94 mmol S/g) and the increases in the C, H and N contents clearly showed the successful synthesis process. The effective amino contents before and after cysteine grafting were about 1.41 and 2.43 mmol U/g, respectively, i.e., the relative ratio to the AMCT was about 1.72 times. The nitrogen content of the nanocomposite was 2.43 mmol N/g (~3.40 *w*/*w*). The amine groups in r-CT were ~5.54 mmol N/g. This indicates that the magnetite fraction was about 50 ± 10%.

The symbolical amine density (=the number of amine molecules/specific surface area (in nm^2^/g)) was about 17.19 for Cys and 713.74 for r-CT (S_BET_: 4.56 m^2^/g for particle size ≤ 100 μm). The N and magnetite contents showed a large dispersion, likely due to the insertion of other atoms. The theoretical content took into consideration the amount of organic reagents used during the synthesis steps.

#### 3.2.2. pH Point of Zero Charge (pH_PZC_) by Drift Titration Method

**Figure 1:**
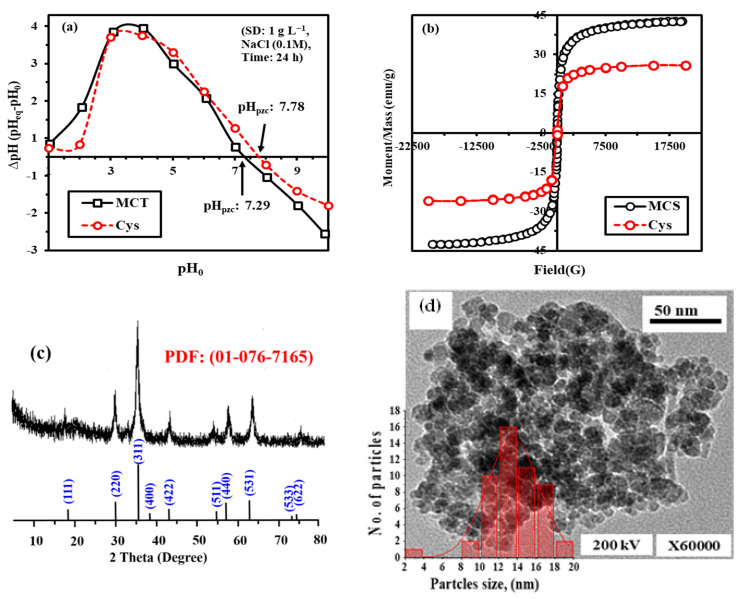
(**a**) Determination of pH_PZC_ by the pH-drift method; (**b**) Magnetization curves of MCT and Cys; (**c**) XRD patterns of Cys (the bar-type spectrum represents the XRD referenced pattern of magnetite); and (**d**) TEM micrographs (the bar is 50 nm).

Figure 1a reports the titration profiles of MCT and Cys, which were consistent with these successive steps. The pH_PZC_, which corresponded to ∆pH = 0, was hardly changed by cysteine grafting on magnetic chitosan: 7.29 ± 0.02 and 7.78 ± 0.02 was recorded for MCT and Cys, respectively. The pH_PZC_ of the MCT particle was affected by the Fe_3_O_4_ core: it was close to 7.29. This was lower than the pH_PZC_ of r-CT: 8.52, and is in line with the pKa value of 6.3–6.6 for the –NH_2_ groups of chitosan biopolymer [13,20]) due to the charge density, screening effect, and amount of impeded Fe_3_O_4_ [20]. After cysteine grafting, the pH_PZC_ increased to 7.78 ± 0.02. This can be easily explained by the immobilization of additional amine, carboxylic acid, and thiolate (-S-) groups. The pK_a_ values of cysteine were 1.71 and 7.85 for the –COOH group and –NH_2_ group, respectively [14], while the cysteinyl thiol group has been found to have different pK_a_ values of 6.4–7.4 [19]. The grafting of cysteine onto the activated magnetite–chitosan nanocomposite appeared to make the net results of all the acid–base properties of the composite (pH_PZC_: 7.78 ± 0.02) closer to the cysteine’s amine moiety against the carboxylic group end.

#### 3.2.3. Magnetic Properties

The magnetic curves of MCT and Cys were recorded and compared using VSM at room temperature (Figure 1b). Obviously, both samples were superparamagnetic and characterized with no hysteresis loop or remanence and coercivity. The MCT magnetic saturation (*M*s) was 42.53 emu/g; however, the raw MCT functionalization (cysteine grafting) reduced the *M*s to 25.81 emu/g. The *M*s values obtained were substantially lower than those recorded from bulk magnetite NPs (~80 emu/g) [25]). For raw MCT, *M*s fell below 42.53 emu/g: magnetite NPs immobilized in polymer matrices showed comparable reductions [25]. The molecular weight of the polymer coating increased according to MCT < Cys, which suggests that the proportion of Fe_3_O_4_ core in the composite reduced in lockstep with the fall in *M*s values [4,25]. Moreover, the *M*s values related to the Fe_3_O_4_ amounts in the MCT and Cys were about 56% and 47%, respectively, from the elemental and mass balance analysis results; the chemical modification increased the organic film thickness and resulted in decreases in the magnetic properties. However, these *M*s values were adequate for facile and effective magnetic separation from the aqueous media.

#### 3.2.4. XRD

The XRD pattern of Cys, which is displayed in Figure 1c, showed eight well-defined diffraction peaks at 2θ = 19°, 30°, 35°, 43°, 54°, 57°, 62°, and 74°, which were indexed to the (111), (220), (311), (400), (422), (511), (440), and (533) planes, respectively. The Fe_3_O_4_ cubic spinel structure is shown by the XRD pattern [26]. However, the potential of maghemite (Fe_2_O_3_) coexisting with ferromagnetic characteristics cannot be ruled out. From XPS, the Fe 2*p* band on the sorbent spectrum can be deconvoluted into three signals (each with its own doublet signal) related to Fe^(III)^ under both octahedral and tetrahedral forms, and Fe^(II)^ (octahedral form). The resolution of the relevant satellite bands was poor. The Fe 2p_3/2_ signal, which was located at 710.6 eV, might have been delegated to the most common types of iron oxides, e.g., Fe_3_O_4_ or g-Fe_2_O_3_ (maghemite) compounds [18]. The size was estimated to be around 12.7 nm using the highest and strongest peak matching to the (311) index (applying the Scherrer equation). This size was systematically (<20 nm) reported for enhanced superparamagnetic character.

#### 3.2.5. TEM Analysis

Figure 1d displays the TEM micrographs of the Cys nanocomposite. The Fe_3_O_4_ nanoparticles appeared as spherical dense dots, surrounded by bright areas, which represent the polymeric shell (due to the electron-absorbing disparities between organic and inorganic components [25]). The nanocomposite had a regular spherical structure, well proportioned, and were apparently mono-dispersed. However, iron oxide nanoparticles (Fe_3_O_4_ NPs) have a tendency to aggregate, owing to magnetic interaction between dipoles/dipoles [5]. This confirmed the successful incorporation of the nanoparticles into the polymeric matrix. The aggregation verified the high magnetism, which corresponded to magnetism measurements. The particle size observed on the TEM images was used to visualize the actual size of the Fe_3_O_4_ NPs. Image software was used to obtain the size distribution histograms counting at least 50 particles, and the average particle size was found to be 14.01 ± 3.7 nm with a narrow particle size distribution (Figure 1d), which was consistent with the XRD results. The size of the Fe_3_O_4_ particles remained <20 nm; this supports the theory that the sorbent can be classified as a nanocomposite.

#### 3.2.6. Textural Properties

**Figure 2:**
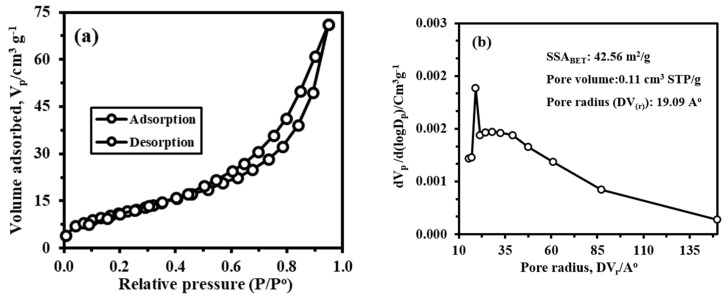
Textural characterization of Cys nanocomposite pore size analysis—N_2_ adsorption/desorption isotherms (**a**) and pore–size–distribution (**b**).

The textural properties of the synthesized Cys nanocomposite were evaluated using the BET equation, which was used to calculate the specific surface areas. Additionally, the pore volume and pore diameter were deduced from N_2_-adsorption–desorption isotherms. The isotherm profile was the type II form, which were classified as nonporous materials according to the Langmuir classification, and they had a wide distribution of pore sizes (Figure 2). The specific surface area was 42.56 m^2^/g, the porous volume was close to 0.11 cm^3^ STP/g, and the average pore size was ~3.82 nm (i.e., pore radius DV(r): 19.09 A°) with a mono-modal (i.e., uniporous) structure distribution. This indicates that the nanocomposite is a mesoporous in nature. BET analysis showed that SSA_BET_ was ~42.56 m^2^/g: this is about 20–30 times the normal value for chitosan flakes, which is consistent with natural and synthesized magnetite values [27]. This value is significantly lower than that expected for nanoparticles: this suggests that the chitosan-based material did not completely cover all iron magnetic nanoparticles. This matches the TEM finding of a thin film of chitosan coating the iron core.

#### 3.2.7. FTIR Analysis

**Figure 3:**
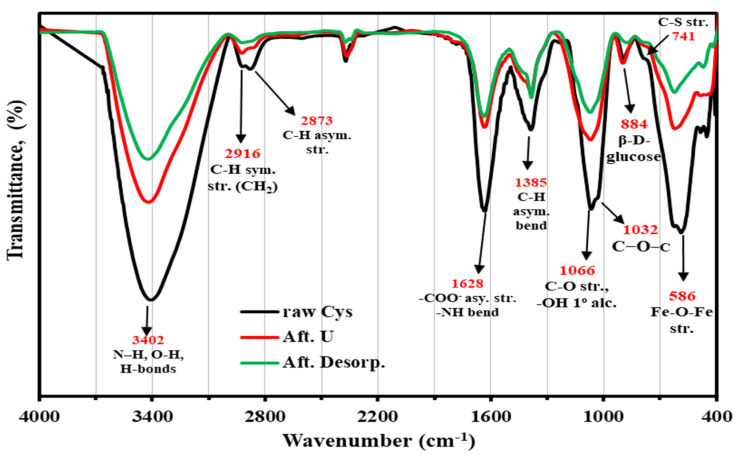
FTIR spectra of the Cys sorbent before and after uranium loading, and at the end of the sixth sorption/desorption cycle.

The functional groups present on the surface of the Cys nanocomposite were identified using FTIR spectrum (Figure 3). Several common bands can be identified. The band at 568 cm^−1^ corresponded to the υ(Fe–O) lattice stretching vibration of magnetite (Fe_3_O_4_) nanoparticles [4]. These two bands were the result of a split in the υ_1_ of the bulk Fe_3_O_4_ (υ(Fe–O)) at 568 cm^−1^, as well as the observed band at ~423 cm^−1^, which was the blue-shift of the bulk magnetite’s υ_2_ (Fe–O bond) [22]. A strong and broad absorption band at 3402 cm^−1^ can be attributed to overlapped bands for –OH and –NH stretching vibrations, as well as polysaccharide hydrogen bonds [22]. The high-intensity peaks in 2916, 2873, and 1384 cm^−1^ were assigned to C–H stretching vibration (CH_2_ at C–6, symmetric and asymmetric) and C–H bending, respectively [7,28]. The significant peak at 1628 cm^−1^ can be attributed to N–H bending vibration (–NH_2_ and >NH amine bend–in plane deformation), as well as symmetric stretching of (COO^−^) carboxylate group grafting [5,7]. The peak around 2363 cm^−1^ might have been due to stretching O=C=O in CO_2_. The two shoulders observed at 1066 and 1032 cm^−1^ were ascribed to C–O–C (in β–glucosidic linkage), C_3_–OH, and/or that of C–C stretching vibration [22]. The bands at 1384 and 1066 cm^−1^ were assigned to primary –OH stretching and secondary –OH stretching, respectively. The band at 884 cm^−1^ represented the β–D–glucose unit [7,20]. The medium/wide band at 468 cm^−1^ was attributed to the out-of-plane bending mode (C–C–N) [29]). The presence of a weak stretching vibration peak of the free C–S functional group at 742 cm^−1^, which vanished after metal sorption, might have been due to its role in metal bonding, which implies that C–S reactive groups play an important role in UO_2_^2+^ binding.

#### 3.2.8. XPS Spectroscopy

**Figure 4:**
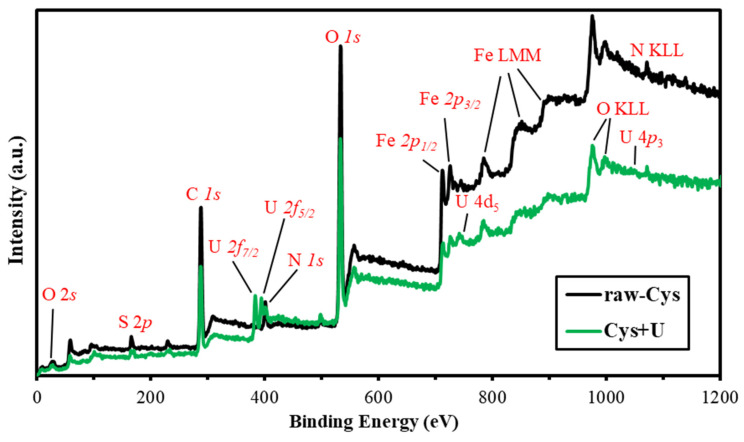

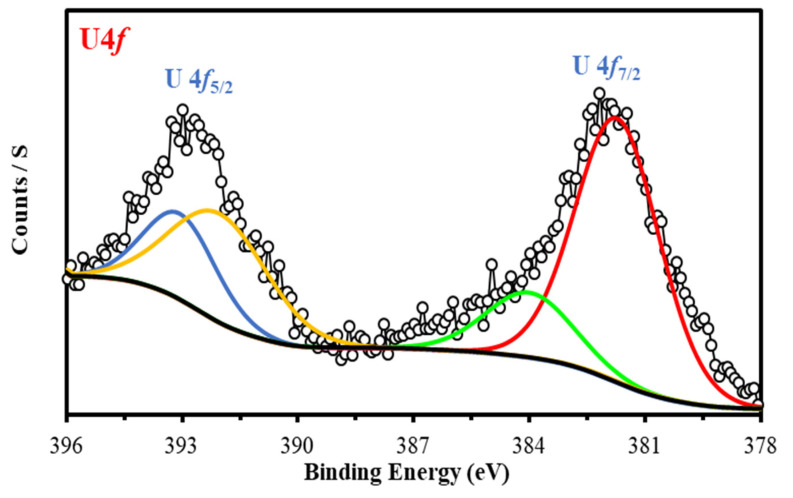
XPS survey spectra of raw Cys sorbent, after U^(VI)^ sorption (**upper panel**) and core level for U 4*f7* (**lower panel**).

XPS analysis was used to determine the chemical compositions of the raw nanocomposite (Cys) and after uranium sorption (Cys + U) (Figure 4). As expected, the XPS spectra of raw Cys revealed C 1*s* (287.7 eV), O 1*s* (533.2 eV), N 1*s* (400.9 eV), and S 2*p* (165.5 eV) signals before and after U(VI) complexation. The characteristic doublets of U 4*f* (hexavalent state) were identified by two peaks around 392.3 eV (U 4*f*_5/2_) and 381.5 eV (U 4*f*_7/2_), which agree well with experimental data spectra [30,31], providing direct evidence of the formation of Cys–U^(VI)^ complexes. Moreover, there are two additional peaks attributed to U element defined as: U 4*d*_5_ and U 4*p*_3_. Furthermore, two peaks at 709–710 eV (Fe 2*p_3/2_*) and 725–726 eV (Fe 2*p*_1/2_) distinguished the magnetite (Figure S1: XPS core level spectra for main elements).

### 3.3. Sorption Studies

#### 3.3.1. Effect of Sorbent Dose

The influence of the sorbent dose (SD: 5–30 mg/20 mL) on uranyl ion sorption and removal efficiencies is shown in Figure S2. The removal efficiency improved from 5 to 30 mg/20 mL, or 0.25 to 1.5 g/L with increases in the sorbent dosage, reaching a maximum (99.5%) at 1.5 g/L; however, the sorption capacity decreased dramatically. This was likely due to the growing number of accessible binding sites. As the sorbent dose was increased, sorbent molecules partly aggregated and formed bridging bonds, reducing the effective surface area and active sites for metal ion binding [32]. Depending on the process’s goal, decontamination of the effluent (lowest residual concentrations needed) necessitates a high sorbent dosage, whereas metal valorization necessitates metal concentration in the sorbent (i.e., lesser sorbent dosages). For the further experiments, a sorbent dosage of 0.5 g/L was chosen as a suitable balance between these two goals.

#### 3.3.2. Effect of pH

**Figure 5:**
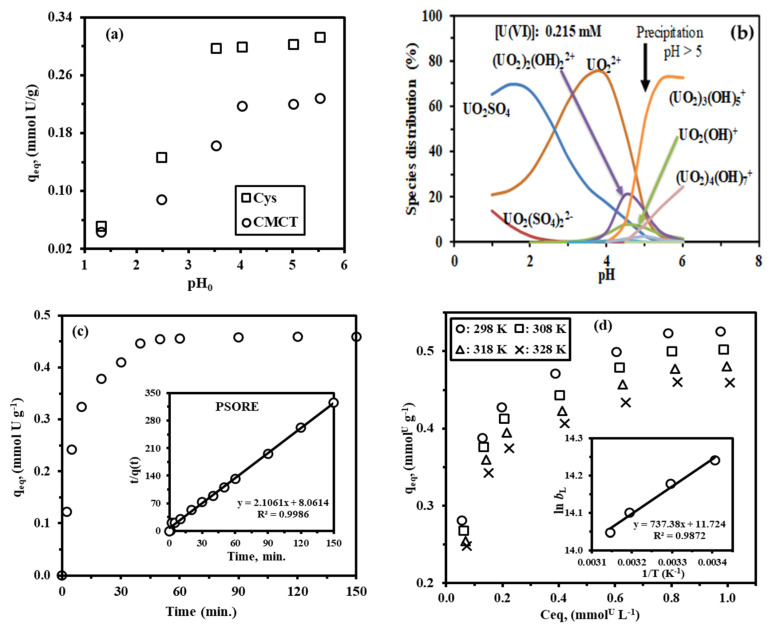
(**a**) The effect of pH on UO_2_^2+^ sorption (C_0_: 0.215 mmol U/L, pH_0_: 1.3–5.5, SD: 0.5 g/L, T: 298 K, Time: 180 min, 200 rpm), (**b**) uranyl speciation (sulfuric acid solution) as a function of pH, calculated by using a Visual–Minted program, (**c**) uptake kinetics and PSORE model (C_0_: 0.43 mmol U/L, pH_0_: 4.0, SD: 0.5 g/L, T: 298 K, Time: 0.0–150 min, 200 rpm), and (**d**) sorption isotherms and thermodynamics: Van ’t Hoff plots of ln *b*_L_ vs. 1/T (C_0_: 0.21–1.26 mmol U/L, pH_0_: 4.0, SD: 0.5 g/L, T: 298–323 K, Time: 60 min, 200 rpm).

Batch experiments at pH_0_: 1.3–5.5 were used to assess the pH impact. The surface charge and target metal ion speciation were determined by the solution pH [12,33]. Figure 5a demonstrates that when the initial and equilibrium pH rose, the sorption capacity increased, particularly between pH_0_ 1.3 and 3.0, before stabilizing at pH_0_ 3.0–5.0 and reaching 0.299–0.303 mmol U/g. When the pH_0_ rose over pH 5.0, the sorption capacity increased slightly, most likely as a result of a shift in metal speciation: when this happens, metal ions begin to produce hydrolyzed products, resulting in a loss of affinity for amino groups, which makes ligand exchange and chelation more difficult [34]. At a lower pH, the sorbent’s surface charge turned positive, and a powerful H^+^ ion competed with UO_2_^2+^ ions for binding sites, reducing the uranyl ion interaction [32,33]. This was confirmed by the pH_PZC_ section, which demonstrated a positively charged surface in this range at pH_0_ < 7.78 due to the deprotonation of reactive groups. Meanwhile, as the pH increased, H^+^ began to be desorbed, and the UO_2_^2+^ hooked up the free binding sites. Hence, the metal sorption increased on the sorbent surface. For cross-linked magnetic chitosan (CMCT) (as a control) the sorption is still at a modest level (from 0.216 to 0.228 mmol U/g) over a wide range of pH_0_: 4.0 to 5.5 (pH_eq_: 4.80 to 5.42), showing that Cys has a higher superiority over CMCT. This can be related to the cysteine moiety incorporation.

Figure S2b shows the pH_0_ variation after uranyl sorption. In acidic medium, the lone pair of the electron of amine groups was protonated, which can contribute to ion exchange with uranyl ion species in the sorption process. Figure S2c shows that the relationship between the log_10_D plot and pH_eq_ was linear (R^2^ = 0.9454 and R^2^ = 0.9761 for Cys and CMCT, respectively), and the slope was 0.2978 and 0.2978, respectively. The line slope was connected to stoichiometric exchange with bound metal ions in ion exchange processes, indicating that the ion exchange process involving uranyl ion species is crucial [4]. Distinct processes are associated with different sorption sites depending on the initial pH_0_. The existence of carboxylic, hydroxyl, thiolate, and amine groups, with their appropriate acid–base characteristics, provide a diverse spectrum of sorbent interactions with UO_2_^2+^ [5]. However, based on the distribution of the species U^(VI)^ hydrolysis and polynuclear hydrolyzed uranium species (Figure 5b), uranyl ions in the suggested system will precipitate at higher pHs and initial uranium concentrations [5,20]. As a result, for further studies, an ideal pH_0_ value of roughly pH: 4.0 was selected.

A multi-linearity for the relationship between plot pH_0_ and q_eq_ was observed (Figure S2), implying that different stages of the mechanism occurred throughout the sorption process. The sorption process takes place in two reactions, ion-exchange (at low pH) and chelation (at slightly acidic and neutral pH) mechanisms (see above). For Cys, the first linear segment had a steep slope (“pH_0_ = 0.1107 q_eq_ + 0.1057” (R^2^: 0.976), and for the second segment, the slop value was nearly relatively close to zero (“pH_0_ = 0.004 q_eq_ + 0.2828” (R^2^: 0.990). This means that in the range pH_0_: 1.3–3.5, the sorption capacities (q_eq_) were strongly changed, while they were not changed at pH_0_: 3.5–5.0, i.e., equilibrium (q_eq_) is reached and stabilizes around this range. This relationship is consistent with the obtained results.

#### 3.3.3. Equilibration Time

According to uptake kinetics, pseudo-equilibrium was attained in 50 min (under specific experimental settings) (Figure 5c). There were several phases in the process that may be identified: (a) in the rapid first stage (during the first 5 min), the sorbent removed roughly 54% of total sorption (q_eq_); (b) the second stage (which can take anywhere between 5 and 30 min) resulted in complementary sorption, which accounted for up to 90% of total sorption; and (c) in the last slower step, at 50 min, the total percentage was 99.78%. The sorbent had mesopores large enough to allow uranium ions to pass through (Shannon radius for hydration [UO_2_(H_2_O)_5_]^2+^ is 1.08 Å [20]), which was in accordance with textural analysis. To attain the actual equilibrium, it is required to wait 50 min.

Figure S3 depicts the modelling of kinetic profiles using a simplified model of intraparticle diffusion resistance (Table S1) [35,36]. There were three linear segments, one for each of the three stages described in Figure S3. Essential characteristics are shown in Table 2. The intraparticle diffusion rate constants’ values (K_id_) for both the first and second rate constants (K_id,1_ and K_id,2_) were higher, and ordered as following: K_id,1_ > K_id,2_, implying the first stage refers to quick sorption on the most accessible active sites on the surface of the Cys nanocomposite (carboxylate, hydroxyl, and amine groups). After that, the concentration gradient between the solution and the sorbent diminished, slowing the mass transfer of uranyl ions to interior reactive groups (the structured material first interior layers and/or larger pores). Finally, the K_id,3_–values were almost nil, as a result of the sorption–desorption equilibrium; it was assumed that sorption occurred in the micropores (the sorbent was nearing saturation) [35,37]. This implies that sorption via very tiny pores is governed by the resistance to intraparticle diffusion [38], as evidenced by a textural analysis.

The PFORE and PSORE were used to model the kinetic behavior (Table S1 for model equations). Heterogeneous reactions are described using the PFORE and PSORE models suggested and constructed for homogeneous chemical processes, under the assumption that apparent rate coefficients are implicitly functions of diffusion resistance [34]. Figure S3 shows the fitting of the experimental data and the parameters listed in Table 2. The PSORE’s experimental data (R^2^ > 0.99) had a substantially higher correlation coefficient than the PFORE’s (R^2^ ~ 0.95). These findings show that the PSORE is an excellent model for understanding uranyl sorption, particularly chemisorption, which involves ion exchange and chelation mechanisms. Furthermore, the PSORE model fitness was validated by virtually the same sorption capabilities (calculated and experimental, Table 2): the PSORE data were overstated by ~4.43%, whereas the PFORE data were underestimated by ~20%. Moreover, the half-sorption time (t_0.5_: the time it takes for the ion exchange resins to attain half of their maximal sorption capacity) was also calculated based on the acquired *k*_2_ values. As mentioned (t_0.5_ = 1/k_2_.q_2_), the t_0.5_ was able to be used to calculate the sorption rate, which was 3.83 min.

#### 3.3.4. Sorption Isotherms

The sorption isotherms (q_eq_ = f(C_eq_)) show how the solute is distributed between the liquid (C_eq_) and solid (q_eq_) phases (for various initial concentrations (C_0_), but for the same temperature and pH) [4,39]. Figure 5d depicts data on UO_2_^2+^ sorption isotherms at pH_0_: 4.0 and various temperatures (298–328 K). The isotherm was characterized by a gradual increase in sorption capacity, followed by sorbent saturation at the maximum experimental sorption capacity of ~0.55 mmol U/g. The plateau revealed that the sorbent’s most active sites reacted with UO_2_^2+^. Furthermore, the underlying curves at various temperatures show that temperature has a detrimental influence on sorption efficiency (i.e., exothermic feature).

Sorption isotherms can be modeled using Langmuir, Temkin, and Freundlich equations (Table S1 for model equations) [37,40,41]. Table 3 shows the computed parameters. The comparison of determination coefficients (R^2^) for the Langmuir equation generally gives slightly higher coefficients (R^2^ > 0.99) compared to the Freundlich (R^2^ < 0.91) (Figure S4). Furthermore, the sorption capabilities matched the experimental data, and both the Langmuir and Freundlich models were persistently overstated (∆q_eq_: 4.6–6.7%). As a result, Monolayer uniform sorption occurred, with a finite number of identical sites scattered over the sorbent surface. Moreover, the Langmuir constant *(b*_L_) was proportional to the sorption energy; the higher the *b*_L_ value, the higher the sorption energy, and the greater the sorbent–sorbate affinity [40]. Table 3 illustrates that both the q_max_ and the *b*_L_ values slightly decreased with increasing temperature, and that the sorption was likely to have been slightly exothermic. The *b*_L_ values decreased (from 17.58 to 14.48) with increases in the temperature (from 298 K to 328 K). This means that at room temperature, sorption is more favorable.

Figure S5 compares UO_2_^2+^ sorption isotherms at pH_0_ 4.0 and C_0_: 1.25 mmol U/g at 298 K for CMCT and Cys: the maximal sorption capacities follow the following order: Cys (~0.546 mmol U/g) > CMCT (~0.291 mmol U/g). This indicates that the chemical functionalization significantly improved the sorption performance by about 1.88 times. This result supports and in agree with the findings of the pH factor.

Another model is the Temkin isotherm, which supposes that sorption free energy is proportional to surface coverage [35,41]. The Temkin model constants are reported in Table 3 and Figure S4. The constant A_T_ represents the sorbent’s initial sorption heat: the higher the A_T_, the greater the sorption heat, and the higher the sorbent’s affinity for the sorbate [41,42]. The A_T_ values reduced from 876.73 to 601.36 L/mmol, and the energetic parameter (A_T_) decreased (from 0.0831 to 0.0769 J/mol) with increases in the temperature from 298 to 328 K, as previously observed (such as in the comparison of *b*_L_ and q_m_ in the Langmuir model) [42].

Based on the Langmuir isotherms (i.e., the *b_L_* constant was employed after conversion (L mg 1 to L mol 1) and dimensionless processing (correcting with the water molality), according to Lima et al. [43]), the thermodynamics can be examined. Thermodynamic parameters are calculated by the Van’t Hoff equation (Equations (12) and (13)) [35,41].

(12) $$\ln b_{L}=-\frac{∆H^{o}}{R}\times \frac{1}{T}+\frac{∆S^{0}}{R}$$

(13) $$∆G^{o}=∆H^{o}-T∆S^{o}$$

where the universal gas constant is R (8.314 J/mol.K), T (K) indicates absolute temperature, and b_L_ is the Langmuir constant. The ∆H^o^, and ∆S^o^ values were obtained from the slope and intercept of the plot of ln *b*_L_ vs. 1/T (Figure 5d).

Table 4 summarizes the thermodynamic parameters. The exothermic nature was confirmed by ∆H^o^’s negative sign, and the reaction was more favorable at lower temperatures, as the ∆H^o^ values were less than 40 kJ/mol, suggesting that physical forces were present in the UO_2_^2+^ sorption process. The positive ΔS^o^ value showed that the interaction between UO_2_^2+^ and Cys would enhance entropy, resulting in more degrees of freedom for solute molecules [33]. The negative ΔG^o^ values (in the same range from −35.18 to −37.61 kJ/mol) imply that the sorption process is spontaneous. The absolute ΔG° values were proportional with the temperature. Moreover, Table 5 indicates that the reaction was more controlled by entropic changes than by enthalpy changes (│ΔH°│ < │TΔS°│) (with values very close).

#### 3.3.5. Comparison of Sorbent Properties for Different Sorbent Material

Compared with other sorption materials, the Cys nanocomposite had an excellent sorption performance, although a direct comparison was difficult due to the differences in experimental settings (Table 5). The nanocomposite sorbent had a good maximum sorption capacity, its equilibrium time was found to be 50 min, and it had a wide range of pH: 3.5–5.0, which indicates that sorbent is the appropriate candidate for uranium sorption.

#### 3.3.6. Metal Desorption and Recycling

**Scheme 2:**
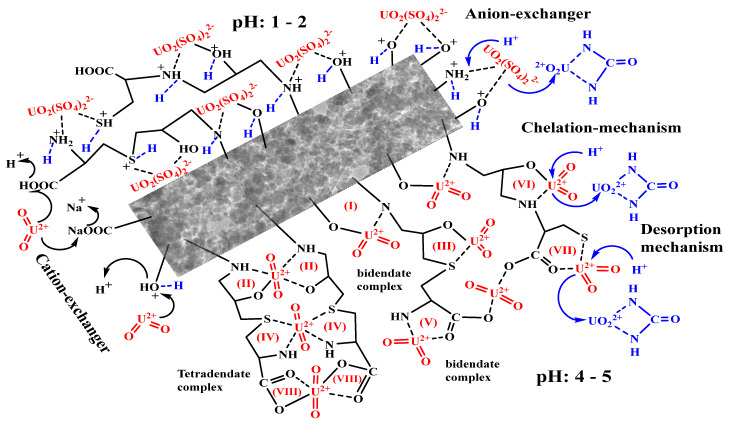
Tentative U^(VI)^ (UO_2_^2+^ and UO_2_(SO_4_)_2_^2−^) sorption interactions modes between reactive groups and desorption from metal−loaded sorbent.

To improve the cost effectiveness, the uranium concentration for final recovery should be improved, and the sorbent should be regenerated and recyclable. Metal desorption from a variety of functionalized resins was particularly efficient with NaHCO_3_ and acidic urea solutions [5,34]. Sorption/desorption were performed repeatedly using acidified urea (0.25 M at pH 2.5) by H_2_SO_4_ as an eluent, and the proposed desorption mechanism was depicted in Scheme 2. Table 6 reports six successive sorption/desorption cycles: both the sorption and desorption efficiencies fell somewhat for each sorption step. At the end of the sixth cycle, the sorption and desorption processes had efficiency losses of less than 9% and 5%, respectively. In terms of sorption capacity, this suggested that the sorbent had strong durability, reusability, and stability.

#### 3.3.7. Uranyl Species Interactions and Mechanisms

The numerous functional groups involved in the uranium sorption process were predicted using extensive spectroscopic analyses (FTIR and XPS).

##### FTIR Spectroscopy

FTIR spectra were used for characterizing the interaction modes and, more specifically, for identifying the reactive groups affected by the binding of UO_2_^2+^ (Figure 3). Basically, the two spectra were almost identical; the significant differences were observed in the form of minor band shifts and decreased peak intensities and broadness in the transmittance spectra when compared, but also in the form of the disappearance of typical bands.

(a) The most notable changes were detected at around 3402 cm^−1^; the broad band (overlapped bands for –OH and –NH stretching vibrations) was little shifted and its width and intensity decreased, as a result of its role in metal ion chelation and sorption.

(b) The bands’ intensity tended to strongly decrease and red-shift at 2916 cm^−1^ and 2873 cm^−1^ (assigned to –CH_2_ groups), moving to 2917 cm^−1^ and 2871 cm^−1^, respectively. These alterations and changes are directly related to metal ion binding, which changes the −OH and −NH groups’ surroundings (band superimpositions that are not well-resolved).

(c) The intensity of bands tended to decrease and red-shift at 1628 cm^−1^ to 1631cm^−1^, which was probably due to the contribution of amine and carboxylate groups, demonstrating a preference for active sites with O- and N-atoms.

(d) The bands at 1385 cm^−1^ and 1066 cm^−1^ (attributable to N-H vibrations in secondary amines and primary/secondary –OH stretching, respectively) moved to 1382 cm^−1^ and 1061 cm^−1^; these changes were associated with UO_2_^2+^−N and UO_2_^2+^−O bonds of N-H and O-H vibrations.

(e) The peak vanished at 742 cm^−1^ (weaker C–S bond stretching vibration), indicating that C–S reactive groups are significant in UO_2_^2+^ binding.

(f) The other significant change (band width and intensity), which was observed at 600–1100 cm^−1^, was affected by metal sorption.

(g) Finally, the absence of characteristic peaks of the linear vibration of UO_2_^2+^ at 823 cm^−1^ and 913 cm^−1^ [34] could not be observed due to it overlapping with other active sites and/or the UO_2_^2+^ contents being too small. However, the XPS analysis verified this.

The desorption spectrum (at the end of the sixth desorption cycle) partly recovered the sorbent, which was exceptionally stable. However, it is noteworthy that many tracer bands of metal sorption remained on the FTIR spectra of the regenerated sorbent, and/or were associated with the effects of environmental conditions (such as pH effect (pH_0_: 4.0)), which can be considered as “intermediary” spectra between raw and metal-loaded sorbents. As seen at the 3402 cm^−1^, 2916–2873 cm^−1^, 1628 cm^−1^, 1385 cm^−1^, 1066–1032 cm^−1^, and 750–400 cm^−1^ regions, the spectrum still had not been fully restored.

##### XPS Spectroscopy

Figure S1 shows the core level spectra for key elements. The deconvoluted bands, via the assignments and relative atomic fractions for the C 1*s*, O 2*s*, N 1*s*, S 2*p*, Fe 2*p*, and U 4*f* signals from HRES spectra for the Cys nanocomposite before and after uranium complexation, are summarized in Table 7. The observed variations in the relative contributions of the different bands and the shift in BEs were mainly followed by U^(VI)^ interaction with N-containing groups (amine), S-containing groups (thiolate/-SH), and O-containing groups (-OH/-COO^−^), as indications of reactive groups’ participation in metal ion binding and/or reactive group chemical alterations.

(a) C 1*s* signal intensities were significantly affected after U^(VI)^ sorption; individual component peaks were identified at 285.36 eV (C–C and C–O), 286.37 eV (corresponding to C–S/C–SH, C–NH and C–OH), and 287.23 eV (C=O and –COO^−^) [50].

(b) The O 1*s* signal showed strong changes after U(VI) sorption; the increase in the band intensity at 531.99 eV corresponding to the C=O and O–C=O function was involved in metal sorption, while decreases at 532.92 eV (C–OH/C–O–C of carbohydrate ring and interchain bonding) occurred due to the environment of these reactive groups [50]. With the inclusion of magnetite (Lattice O signal for magnetite Fe–O), a band at 530.7 eV (of constant intensity) developed

(c) N 1*s* appeared after cysteine grafting; the band’s relative intensity at 400.9 eV (–NH_2_, >NH) was increased with a shift in BE to 400.90 eV (where secondary amine (>NH) from chitosan moiety and primary amine (–NH_2_) from cysteine moiety were involved in uranium sorption). Meanwhile, that of C–N and N–H, as well as N-U (399.5 eV), decreased.

(d) The S 2*p* signal confirmed the occurrence of S-atom in the Cys sorbent as an organic form. The asymmetric S 2*p* peak was deconvoluted into two subpeaks assigned to S 2p_3/2_ and S 2p_1/2_, which were noticed at 164.17 eV and 168.05 eV, respectively, indicating the S^2−^ presence as C–S–C [51]. There was a relative decrease in their intensity, indicating that S-atom is involved in U sorption as a chelation thiolate-U via S-orbitals. Hamza et al. [52] reported that metal ion sorption is mainly followed by: (a) changes in the relative contributions of the various bonds, and (b) shifts in BEs, as tracers of the involvement of reactive groups in the binding of metal anions, and/or the chemical modifications of reactive groups (associated with the reduction of U(VI) and the oxidation of the surface groups on the sorbent). Surprisingly, a new peak arose at 169.43 eV after U^(VI)^ sorption, confirming the existence of inorganic sulphate anions (SO_4_^2−^) immediately on an ion pair (protonated amine groups) at the sorbent’s surface. This was likely owing to the sorption of uranyl sulfate forms (speciation of uranyl anions from sulfate medium), confirming that anion-exchange mechanisms were involved (See Scheme 2 below).

(e) The Fe 2*p* signal showed the primary peaks of Fe^3+^ 2*p*_3/2_ and Fe^3+^ 2*p*_1/2_, which were regarded to be unique Fe_3_O_4_ spectra, and these were situated at 711.18 and 724.31 eV, respectively [53,54]. By fitting the Fe 2*p* double peak, the Fe 2*p*_3/2_ peaks emerged at 710.68 and 713.23 eV, and the Fe 2*p*_1/2_ peaks appeared at 719.99 and 724.31 eV, respectively, confirming the existence of Fe^2+^ and Fe^3+^, respectively, corresponding to Fe_3_O_4_ [54]. A Fe 2p_3/2_ satellite and Fe 2p_1/2_ satellite appeared at 718.2, and 732.39 eV, respectively [54,55]. However, Fe_3_O_4_ nanoparticles and Fe^2+^ shakeup satellite peaks (at 716.96 eV) can be observed beside the Fe^3+^ shakeup satellite (at 719.99 eV) due to Fe atoms migrating to the surface and the ease of identification by XPS [54,55]. After sorption, no impressive changes were observed (Figure S1: Fe 2*p* signal), because Fe atoms are at the structure’s core and the sorption of UO_2_^2+^ has no affect them [54].

(f) For U 4*f* excitations, deconvoluting core level spectra into two main spin–orbits (L–S) split the U 4*f*_7/2_ and U 4*f*_5/2_ signals at about ~381.65 and ~392.26 eV, respectively, consistent with previously reported spectra [30,31]. Inspection of the satellite peaks showed the occurrence of peaks at 4 and 7 eV from the U 4*f*_7/2_. Shake-up satellites are low-intensity excitations that arise as a result of the photoelectron excitation process’s shift in electrostatic potential [30]. The core level electron kinetic energy will drop as a result, and an additional/satellite peak will form on the high binding energy (BE) side of the main peak [30,31].

#### 3.3.8. Metal Interaction Mechanism

Based on the effect of pH results, FTIR and XPS data show that distinct strategies were used in uranium sorption (Scheme 2):

(a) Anion-exchange mechanism of anionic UO_2_(SO_4_)_2_^2−^ species via SO_4_^2−^ on the protonated amine groups;
(b) Cation-exchange mechanism with cationic species (UO_2_^2+^) at higher pH_0_, but less than pH_PZC_ (i.e., can deprotonate active sites), with the Na^+^ attached to –COO^−^ and H^+^ of the –OH and –NH_2_/>NH groups;
(c) Chelation and complexation mechanisms of UO_2_^2+^, with different heteroatoms (O and N) on the surface of the sorbent.

The chelation and complexation interaction mechanism of U^(VI)^ with Cys, via various O-, S-, and N-atoms, produced metal–ligand interactions. This was clarified with XPS findings at the molecular level by comparing the raw Cys before and after U^(VI)^ loading. The high-resolution C 1*s* spectra (Figure S1) suggest some differences between the C 1*s* peak for the raw Cys before and after sorption of U^(VI)^. Significant differences (both in intensities and peak positions) can be observed for the S 2*p*, N 1*s*, and O 2*s* signals, which are associated with sulfur-, nitrogen-, and oxygen-containing functional groups, such as the –S– in the thiolate linker, the –SH thiolate group (via amine linked with cysteine), >NH in the chitosan molecule, terminal –NH_2_ bonds in the cysteine group, >C=O, –COO^−^ of the cysteine moiety, and –OH in the chitosan molecule in a single sorbent chain.

A putative mechanism for the uranyl metal ion complex may be hypothesized based on XPS and FTIR investigations (Scheme 2). Metal ions were bound via the following mechanisms: (a) two coordinating bonds with the N-atom of >NH group, the O-atom of primary C4-OH in the chitosan molecule itself (the contribution modes I), and with the secondary (–OH) from the epichlorohydrin binder between chitosan and the cysteine moiety (the contribution modes II); (b) two coordination bonds with the O-atom of secondary (–OH) from the epichlorohydrin binder primary hydroxyl group, and the S-atom in the thiolate linker of the cysteine moiety (the contribution modes III); (c) two coordination bonds with S-atom of the thiolate linker and the N-atom of the amine group in the cysteine moiety (the contribution modes IV); (d) two coordination bonds with the O-atom of (>C=O) carbonyl group, the N-atom of the amine group in the cysteine moiety (the contribution modes (V)); and, finally, (e) two coordination bonds with oxygen atoms of the carboxylate group, forming four membered rings. Another mode of cysteine-linking to the matrix via amine group was observed, as explained above (Section 3.1), so it had a free terminal thiol-group (−SH) that could give contribution modes VI and VII). Thus, contribution modes I to VI and VII formed stable five- and six-membered chelating rings, respectively, whereas modes VIII formed an unstable four-membered ring. Moreover, another interaction mode between UO_2_^2+^ and active sites from two chains formed two chelating rings (with five members in size). By increasing the number of chelate rings, the formed complex’s stability improved, and the metal sorption capacity improved as a result (Scheme 2).

Moreover, the anion-exchange mechanism of uranium sorption by amine groups [56], and the suspected anion-exchange reactions were poorly probable: The overall ion-exchange reaction (Equations (14) and (15)):

(14) $${\left(R_{2}{\mathrm{NH}}_{2}^{+}\right)}_{2}{\mathrm{SO}}_{4}^{2-}+{\mathrm{UO}}_{2}{\left({\mathrm{SO}}_{4}\right)}_{2}^{2-}↔{\left(R_{2}{\mathrm{NH}}_{2}^{+}\right)}_{2}{\mathrm{UO}}_{2}{\left({\mathrm{SO}}_{4}\right)}_{2}^{2-}+{\mathrm{SO}}_{4}^{2-}\;$$

Another ion-exchange mechanism for the neutral uranium sulfate species:

(15) $${\left(R_{2}{\mathrm{NH}}_{2}^{+}\right)}_{2}{\mathrm{SO}}_{4}^{2-}+{\mathrm{UO}}_{2}{\mathrm{SO}}_{4}↔{\left(R_{2}{\mathrm{NH}}_{2}^{+}\right)}_{2}{\mathrm{UO}}_{2}{\left({\mathrm{SO}}_{4}\right)}_{2}^{2-}\;$$

Finally, it is noteworthy that the proposed desorption process, depicted in Scheme 2, used acidified urea in the desorption stage, as the acidity of this solution can facilitate and break the contribution modes; in addition, urea can coordinate and bond with two N-donors of –NH_2_ in the urea CO(NH_2_)_2_ molecule; this confirms the expected mechanism.

#### 3.3.9. Tests of Selectivity

**Figure 6:**
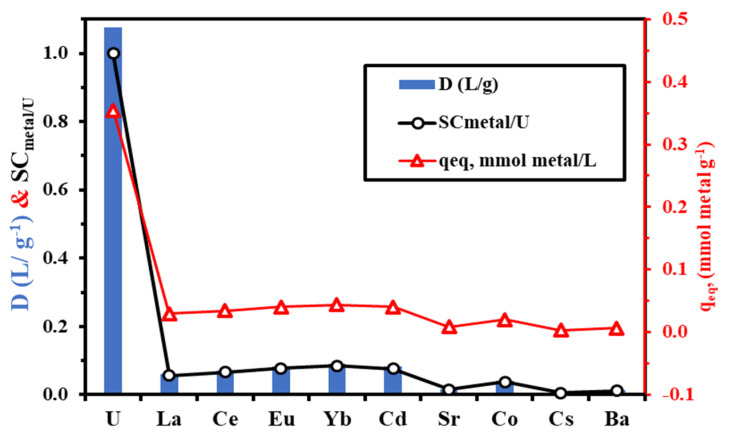
Sorption capacities, distribution coefficients, and selectivity coefficient (SC_metal/U_) in selectivity tests (SD: 0.5 g/L, C_0_: 0.5 mmol/L, pH_0_: 4.0, T: 298 ± 1 K, Time: 180 min, 200 rpm).

The selectivity sorption of UO_2_^2+^ from a simulated nuclear industrial effluent (in multi-component equimolar solutions) was examined. In terms of sorption capacities (q_eq_, mmol/g), the distribution ratio (D, L/g), and the selectivity coefficient (SC_metal/U_ = D_metal_/D_U_) [4], the data were analyzed (Figure 6). The uranium sorption capacity reached 0.354 mmol U/g, which was significantly lower than that of synthetic solutions (0.546 mmol U/g). This suggests that excessive concentrations of co-ions lower maximum sorption by 35.17%; considering the significant abundance of coexisting ions, the sorbent has a strong preference for UO_2_^2+^. Despite the solution complexity, potential changes in metal speciation (due to nitrate anion) and the possible competitive impact of other metal ions should be noted. Other co-metal ions attached to the particular reactive sites through various stoichiometric ratios and/or distinct functional groups, resulting in a total sorption capacity of 0.580 mmol metal/g greater than that of UO_2_^2+^ in synthetic solutions. The distribution ratio (D: q_e_/C_e_) and the selectivity coefficients (SC_metal/U_) were calculated, which highlighted more efficiently the selectivity for the target metal. Hence, U^(VI)^ had higher D values (1.08 L/g) and SC_metal/U_ values than other co-ions; the sorbent, thus, has preferential affinity and competitiveness for highly selective uranium sorption.

**Figure 7:**
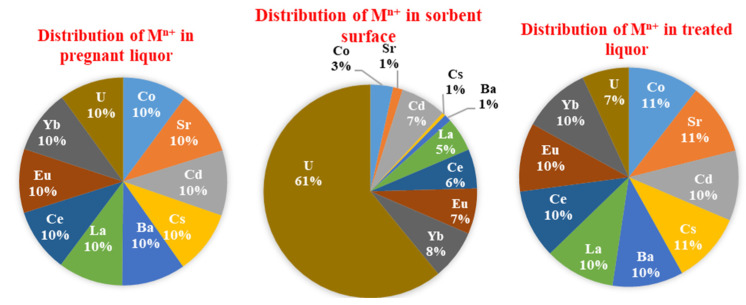
The distribution of the selected metal ions in pregnant liquor, treated liquor, and the Cys-sorbent (molar percentages).

Figure 7 depicts the relative molar percentages of various co-metal ions in the initial tested pregnant liquor, treated liquor raffinate, and the metal ions adsorbed onto the surface of Cys sorbent. There was a significant difference in the solution before and after sorption. The residue concentration of uranium after the sorption process was about 7% molar percent and the sorbent. This is the first proof that the selectivity is quite high for uranium. In comparison to the leachate’s original composition, the percentages in the sorbent reflect a wide range of trends: UO_2_^2+^ exhibited a significant enrichment and concentration (from 10.0% to 61%), as well as to a lesser extent for ƩREEs’ 149.4 µmol RE/g; 85.1µmol HREs/g represented ~58% of the total capacity (i.e., 44.9 and 40.1 µmol/g for Yb^(III)^ and Eu^(III)^, respectively), and 64.4 µmol LREs’ g^−1^ represented ~42% of the total capacity (i.e., 34.5 and 29.9 µmol/g for Ce^(III)^ and La^(III)^, respectively). Alternatively, the sorption capacity of other metal ions represented ~13.5% of the total sorption capacity of ≈78.1 µmol/g, including 40.4 µmol/g of Cd^(II)^, which represented about 51.8% of the total other metal ions, and about 7% of the total sorbent sorption capacity. The selectivity for Cd^(II)^ over other metal ions was likely related to the existence of functional groups that are S-bearing (soft base), which are more sensitive to soft acids such Cd^(II)^ [4], lowering the Cys sorbent overall selectivity for UO_2_^2+^. Moreover, the poor sorption efficiency for both Sr(II) and Cs(I) may be explained by the fact that they have a greater affinity for the ion-exchanger inorganic sorbents than the organic chelation sorbents [57]. Moreover, the QSAR (quantitative structure activity relationship) tools were applied for correlating the sorption properties and selectivity coefficients of the sorbent for the co-metal ions to their specific physicochemical characteristics (e.g., atomic number, electronegativity, and hydrated ionic radius), in order to explain the high selectivity of the uranium over other co-metal ions in the liquor.

Rashad et al. (2021) [42] stated that various factors may regulate the preference of uranyl ions for different sorbents:

(a) According to Pearson’s principles (the HSAB concept), uranyl is a hard acid, which indicates that the metal ion binds preferentially to ligands containing O >> N >> S [58];
(b) The electron-withdrawing/releasing effect affects the donating ability of the different functional groups (negatively for carboxylic acid groups, positively for amine groups);
(c) Uranium is present in oxycation form (UO_2_^2+^), which can be adsorbed in different forms with different mechanisms, as reported in our study;
(d) The steric effect modulates the stability and accessibility to reactive groups: herein, the contribution modes involved sulfur atoms, forming stable five- and six-membered chelating rings, while in other modes forming four membered rings (i.e., stronger steric hindrance when compared with five-/six-membered rings).

**Figure 8:**
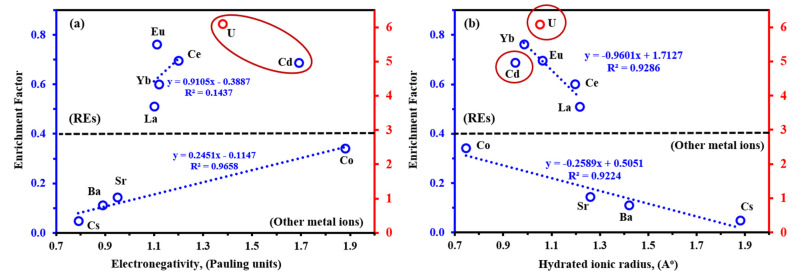

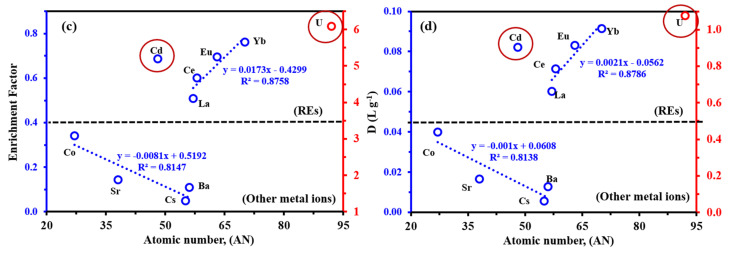
Correlation between atomic number and (**a**) enrichment factor and (**b**) distribution coefficient (D). Enrichment Factor and electronegativity (**c**) and hydrated ionic radius correlations (**d**).

The relationships between the atomic number, the enrichment factor (EF = molar percentage ratio on the sorbent relative to that in the leachate [39]), and the distribution coefficient (D) are shown in Figure 8a,b. The figure reveals obvious trends: the enrichment factor for U(VI) exceeded 6, and raised as the atomic number increased; the same can be observed for the Cd(II), while heavy rare earth ions (HREs) were over light rare earths (with EF > 0.5), and the final other co-metal ions demonstrated a reciprocal tendency. Obviously, the linear curves clearly exclude uranium: U is commonly connected with REEs, due to its atomic number (92). However, the EF order of magnitude was the same as that of HREs. The distribution coefficients, which were much greater for HREEs than for LREEs when compared to other co-ions in the solution, showed the same results. This means that for the REs, both EF and D increased with increases in the atomic number, contrary to the behavior of the other metal ions (i.e., they followed a reciprocal trend). Comparing the chemical characteristics of selected metal ions in Table 8 reveals that the U element was extremely similar to RE elements in terms of ionic radii and electronegativity (Pauling units), but differed significantly in term of atomic mass. The U point shifted to its “equivalent atomic number” deviated significantly from the REs’ linear trends.

Figure 8c,d depicts the relationship between the enrichment factor for REs and other co-metal ions’ electronegativity and hydrated ionic radii. Generally, the hydrated ionic radius was inversely proportional with the enrichment factor after the exclusion of border elements (U and Cd), with little or no enrichment factor change observed. For REs (Yb, Eu, Ce, and La elements), the electronegativity had an irregular/did not contribute on the enrichment factor. Meanwhile, the ER of other metal ions improved and raised with electronegativity, and reduced as the hydration ionic radius increased.

## 4. Conclusions

In this study, a hybrid mesoporous magnetic chitosan nanocomposite was successfully functionalized by cysteine to prepare a Cys sorbent for the sorption of uranyl ions selectively and efficiently from aqueous solution and radioactive effluents. A wide variety of analytical methods (including the CHNS, XRD, BET, pHpzc, TEM, XPS, FTIR, and VSM analyses) were used to determine the nanocomposites’ physiochemical characteristics. The TEM micrograph determined the actual size of Fe_3_O_4_-nanocomposite and the morphology as well. The Fe_3_O_4_ NPs average particle size was found to be 14.01 ± 3.7 nm with a narrow size distribution, according to the obtained histogram with a regular spherical dot structure. The mesoporus sorbent had large surface-to-volume ratios (~42.56 m^2^/g and 0.11 cm^3^/g, respectively), and a unipore structure (~3.6 nm). Batch experimental studies showed that the sorption equilibrated within 50 min, sorbent dose 0.5 g/L, and the sorption capacity was ~0.575 mmol U/g, at 298 K, over a wide range of pH_0_: (3.5–5.0). The sorption complied with pseudo-second-order and Langmuir models, implying that the sorption process tended to be a monolayer chemical sorption process. The comprehensive investigation (FTIR and XPS analysis) of surface interaction speculation found that thiolate, amino, and hydroxyl functional groups were identified as the main reactive groups that absorbed UO_2_^2+^ on the surface of sorbents through ion exchange, electrostatic attraction, and chelation mechanisms. Thermodynamic parameters illustrated that the reaction exhibited spontaneous and exothermic behavior. An acidic urea solution (0.25 M, pH_0_: 2.5) can be used to elute uranyl ions from metal-loaded sorbent. After six successive sorption/desorption cycles: the efficiency of the sorption and desorption processes were less than 9% and 5%, respectively. Competitive sorption–desorption experiments for UO_2_^2+^ showed that the sorbent had considerably efficient sorption, durability, and reusability. U^(VI)^ showed higher selectivity coefficient and distribution ratio values (1.08 L/g) than other co-metal ions, indicating that the sorbent was efficient in the selective recovery of the uranium from simulated nuclear waste solution. The superparamagnetic properties of the nanocomposites (*M*_s_: ~25.80 emu/g) indicate that the sorbent has broad prospects for use in removing uranium from solution.

## Figures and Tables

**Table 1:** Elemental analysis of nanocomposite synthesis stages.

Materials	C (%)	H (%)	N (%)	N (mmol/g)	S (%)
**r-CT**	39.97	6.86	7.76	5.54	---
**MCT**	18.91	4.07	3.58	2.56	---
**CMCT**	18.35	3.93	3.01	2.15	---
**AMCT**	15.82	3.23	1.98	1.41	---
**Cys**	20.51	4.01	3.40	2.43	3.01

**Table 2:** Uptake kinetics for UO_2_^2+^–parameters for the PFORE, PSORE, and sRIDE Model.

Model	Parameters	Values
q_max, Exp._	(mmol U/g)	0.455
PFORE	K_1_ × 10^−4^ (min^−1^)	748.478
	q_max,cal._ *	0.362
	R^2^	0.953
PSORE	K_2_ × 10^−4^ (g/mmol.min)	550.230
	q_max,cal._*	0.475
	R^2^	0.998
sRIPD	K_id,1_ **	0.1051
	K_id,2_	0.0361
	K_id,3_	0.0009

* Units: (mmol U/g), and ** K_id,x_: mmol/g.min^0.5^.

**Table 3:** Sorption isotherms for UO_2_^2+^–the Langmuir, Freundlich, and Temkin equations all include model parameters.

T (K)	q_max.exp._ *	Langmuir Isotherm			Freundlich Isotherm			Temkin Isotherm		
		q_max.cal.,_ *	*b*_L_ (L/mmol)	R^2^	1/n	*K_F_* *	R^2^	A_T_ (L.mmol)	B_T_ (J/mol)	R^2^
298	0.546	0.576	17.58	0.9996	0.1963	0.576	0.933	876.731	0.0831	0.970
308	0.522	0.553	16.49	0.9993	0.1987	0.549	0.914	789.342	0.0806	0.956
318	0.501	0.532	15.25	0.9993	0.2051	0.526	0.905	640.533	0.0796	0.949
328	0.480	0.512	14.48	0.9991	0.2052	0.502	0.9264	601.364	0.0769	0.964

* Units: (mmol U/g).

**Table 4:** Thermodynamic parameters for UO_2_^2+^ sorption.

Temp. (K)	∆H° (kJ/mol)	∆S° (J/mol)	∆G° (kJ/mol)	T∆S° (kJ/mol)	R^2^
298	−6.131	97.473	−35.18	29.05	0.9872
308			−36.15	30.02	
318			−37.13	31.00	
323			−37.61	31.48	

**Table 5:** Uranium sorption performances using different sorbents.

Sorbent	pH_0_	T_eq_ (min)	SD (g/)L	Temp. (K)	q_m_,_exp_ *	q_m,L_ *	b_L_ (L/mmol)	Ref.
HCPs	8.0	70	1.0	308	0.196	0.200	14.520	[33]
SIL	4.0	60	1.0	325	0.168	0.157	19.753	[44]
Arg-Cell Glu-Cell	5.0	180	0.5	318	0.581 0.676	0.618 0.705	17.852 24.993	[12]
CA CA-PO_4_	5.5	120	1.0	298	0.431 0.631	0.431 0.631	26.183 52.366	[45]
DETA-MC	4.0	60	0.2	298	0.748	0.778	22.137	[5]
HA-MCNPs	5–7	20	1.0	333	46.3	47.9	3.166	[46]
Mag. Schiff base	6.0	360	0.02	298	0.396	0.416	138.176	[47]
Schiff’s base-MC	4.0	20	1.0	298	2.322	2.454	1.800	[48]
HTC-Acy	4.5	120	1.0	335	1.716	---	---	[49]
Cys	4.0	50	0.5	298	0547	0.576	17.58	Here

* Unit: mmol U/g.

**Table 6:** Metal desorption and sorbent recycling.

Cycle No.	Sorption Step			Desorption Step		
	SE (%)		qeq (mmol U/g)	DE (%)		Eluate Conc. (mmol U/L)
	Average	St. Dev.	Average	Average	St. Dev.	Average
1	100 *	0.00	0.515	97.88	0.19	0.504
2	97.11	0.08	0.500	96.77	0.26	0.498
3	95.65	0.17	0.492	95.05	0.48	0.480
4	93.81	0.06	0.483	95.43	0.86	0.476
5	92.36	0.25	0.475	94.07	0.50	0.454
6	91.52	0.05	0.471	93.00	0.76	0.442
Δ(1st/6th)	−8.48			−4.88		

Sorption efficiency (SE) and desorption efficiency (DE). * Standard metal ion sorption efficiency (at first cycle).

**Table 7:** Assignment of the core–level signals: Binding energies (BEs) and atomic fraction (AF, %).

Signal	Bef. U–Sorption		Aft. U–Sorption		Possible Chemical Assignments
	BE (eV)	AF (%)	BE (eV)	AF (%)	
C *1s*	285.36	22.88	285.84	35.89	C–H, C–C
	286.37	24.32	286.5	46.5	C–S, C–N, C–O, C–O–C,
	287.23	52.8	288.24	17.57	C=O, COO^-^
O *1s*	530.17	20.29	530.4	20.85	Lattice O (Fe–O, Fe_3_O_4_)
	531.99	42.76	532.53	59.27	C=O, O-C=O
	532.98	36.95	533.14	19.88	C–OH, H_2_O, C–O–C, Metal(OH)_x_
N *1s*	399.54	64.37	399.41	30.59	C–N
	401.36	35.63	400.9	69.41	–NH_2_, >NH
S *2p*	164.17	82.27	164.17	59.11	C–S (*2p_3/2_*)
	168.05	17.73	167.98	14.43	C–S–C (S *2p_1/2_*)
			169.43	26.47	S–O (S 2*p*_3/2_ Sulfate)
Fe *2p*	710.68	31.18	710.92	42.75	Fe *2p_3/2_* (Fe^3+^–octa)
	713.23	19.42	713.45	16.2	Fe *2p_3/2_* (multipl–Fe^2+^–octa)
	716.96	7.97	717.02	5.62	Fe *2p_3/2_* (Fe^3+^–tetra)
	719.99	8.72	720.02	4.25	Fe *2p_1/2_* (Fe^3+^–Satallite)
	724.31	19.88	724.1	17.36	Fe *2p_1/2_* (Fe^2+^–octa)
	727.69	7.8	726.91	9.48	Fe *2p_1/2_* (Fe^3+^–tetra)
	732.39	4.97	733.21	4.34	Fe *2p_3/2_* (Fe^3+^–octa)
U 4*f*			381.82	58.44	U 4f*_7/2_* (U^(VI)^)
			385.61	6.12	U 4f*_7/2_* (U^(VI)^ –Satellite)
			392.12	22	U 4f*_5/2_* (U^(VI)^)
			393.07	13.44	U 4f*_5/2_*(U^VI^ –Satellite)

**Table 8:** Chemical properties of some selected metal ions (under experiments).

Element	Atomic Number	Hydrated Species	M-O Bond Distance (Å)	M^n+^ Ion Radius (Å) [59]	M^n+^ Ion Radius, (Å) [58]	Electronegativity (Pauling Units)
Co	27	Co(H_2_O)_6_^2+^	2.08	0.74	0.745	1.88
Sr	38	Sr(H_2_O)_8_^2+^	2.62	1.28	1.26	0.95
Cd	48	Cd(H_2_O)_6_^2+^	2.3	0.96	0.95	1.69
Cs	55	Cs(H_2_O)_12_^+^	3.25 *	1.91 *	1.88	0.79
Ba	56	Ba(H_2_O)_8_^2+^	2.82	1.48	1.42	0.89
La	57	La(H_2_O)_9_^3+^	2.52 + 2.64	1.18 + 1.30	1.216	1.1
Ce	58	Ce(H_2_O)_9_^3+^	2.54	1.2	1.196	1.12
Eu	63	Eu(H_2_O)_9_^3+^	2.425	1.085	1.062	1.2
Yb	70	Yb(H_2_O)_8.5_^3+^	2.32	0.98	0.985	1.11
U	92	U(H_2_O)_9_^4+^	2.42	1.08	1.05	1.38
		UO_2_(H_2_O)_5_^2+^	(1.70) + 2.42	1.08		1.38

* Information based on solid-state structures.

## Data Availability

All data generated or analyzed during this study are included in this article.

## Supplementary Materials

The following supporting information can be downloaded at: https://www.mdpi.com/article/10.3390/polym14132568/s1, Table S1. Sorption modeling: kinetics and sorption isotherms. Figure S1. XPS core level spectra for C 1*s*, O 1*s*, N 1*s*, P 2*p*, Fe 2*p*, and U 4*f* for Cys-sorbent before and after UO_2_^2+^ sorption. Figure S2. Effect of sorbent dosage (a), Plot of pH0 vs. pH_eq_ (b), log_10_D plot vs. pH_eq_ (b), and plot of pH_0_ vs. q_eq_ (c) for UO_2_^2+^ sorption. Figure S3. Sorption kinetics for PFORE, PSORE, and Intraparticle diffusion. (C_0_: 0.425 mmol U/L, SD: 0.5g/L, time 60 min, room temp. 298 K), and Figure S4. Linearization plots of Langmuir (a), Freundlich (b), and Timken isotherm (c): for UO_2_^2+^ sorption. (pH_0_: 4.0, SD: 0.5 g L^−1^, C_0_: 0.21–1.26 mmol U/L; T: 298–328 K, Time: 60 min.). Figure S5. Sorption isotherm using both CMCT and Cys nanocomposites for UO_2_^2+^ sorption. (pH_0_: 4.0, SD: 0.5 g/L, C_0_: 0.21–1.26 mmol U/L, T: 298–328 K, Time: 60 min for Cys and 180 min for CMCT).
