# Supplementary material for: Mesoporous Magnetic Cysteine Functionalized Chitosan Nanocomposite for Selective Uranyl Ions Sorption: Experimental, Structural Characterization, and Mechanistic Studies
Source: Polymers (Basel). 2022 Jun 24;14(13):2568. doi: 10.3390/polym14132568 (PMC9268972; doi:10.3390/polym14132568)
Supplement: Supplementary file 1 [file polymers-14-02568-s001.zip › polymers-1749332-supplementary.pdf]

Mesoporous Magnetic Cysteine Functionalized Chitosan Nanocomposite for Selective Uranyl Ions Sorption: Experimental, Structural Characterization, and Mechanistic Studies

Ahmed A. Al-Ghamdi [1], Ahmed Galhoum [2] Ahmed Alshahrie [1,3], Yusuf A. Al-Turki [4], Amal M. Al-Amri [5], S. Wageh [1,*]

[1] King Abdulaziz University, Faculty of Science, Department of Physics, Jeddah, Saudi Arabia; agamdi@kau.edu.sa
[2] Nuclear Materials Authority, P.O. Box 530, El-Maadi, Cairo, Egypt; Galhoum_nma@yahoo.com
[3] Centre of Nanotechnology, King Abdul-Aziz University, Jeddah, Saudi Arabia; aalshahri@kau.edu.sa
[4] King Abdulaziz University, Faculty of Engineering, Computer and Electrical Engineering Department, Jeddah, Saudi Arabia; yaturki@yahoo.com
[5] King Abdulaziz University, Faculty of Science & Arts, Physics Department, Rabigh, Saudi Arabia; amsalamri@kau.edu.sa
* Correspondence: wswelm@kau.edu.sa; (S. Wageh)

Supporting Information section

Table S1. Adsorption modeling Equations: kinetics and isotherms [1, 2]

Process	Model	Equation	Parameters	
Kinetics	PFORE	$q(t) = q_{eq,1}(1 - e^{k_1 t})$	$q_{eq,1}$ (mmol^U/g)	k_1 (min^{-1})
	PSORE	$q(t) = \frac{q_{eq,2}^2 \times k_2 \times t}{1 + q_{eq,2} \times k_2 \times t}$	$q_{eq,2}$ (mmol^U/g^{-1})	k_2 (L mmol^{-1} min^{-1})
	sRIDE (Weber & Morris)	$q_{(t)} = k_{int.,i} \cdot t^{0.5} + C$ Several linear sections corresponding to different regimes of resistance (i) to intraparticle diffusion may co-exist ($K_{int,\,i}$) (linear regression calculation)	$K_{int.,i}$ (mmol g^{-1} min$^{-0.5}$)	
Isotherms	Langmuir	$\frac{C_{eq}}{q_{eq}} = \frac{C_{eq}}{q_{max}} + \frac{1}{b q_{max}}$	q_{max} (mmol^U/g)	b_L (L/mmol)
	Freundlich	$q = k_F C_{eq}^{1/n}$	k_F	N (dimensionless)
	Temkin	$q_{eq} = B_T \ln C_{eq} + B_T \ln A_T$ Where, $B_T = \frac{RT}{b_T}$	A_T (L/mmol)	b_T (J/mol)

Figure S1. XPS core level spectra for C 1*s*, O 1*s*, N 1*s*, P 2*p*, Fe 2*p*, and U 4*f* for Cys-sorbent before and after UO_2^{2+} sorption.

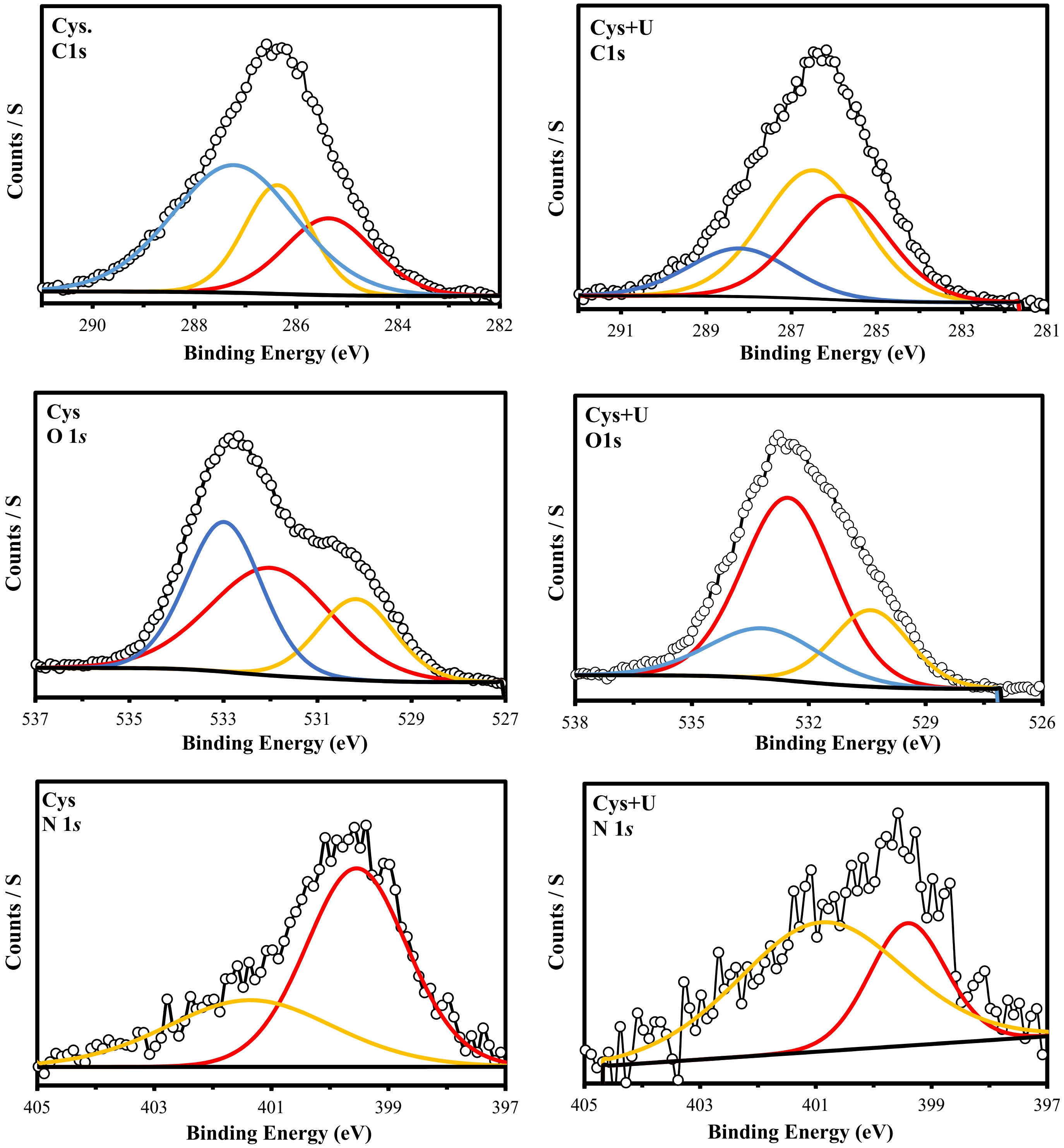

Figure S1. (*cont.*)

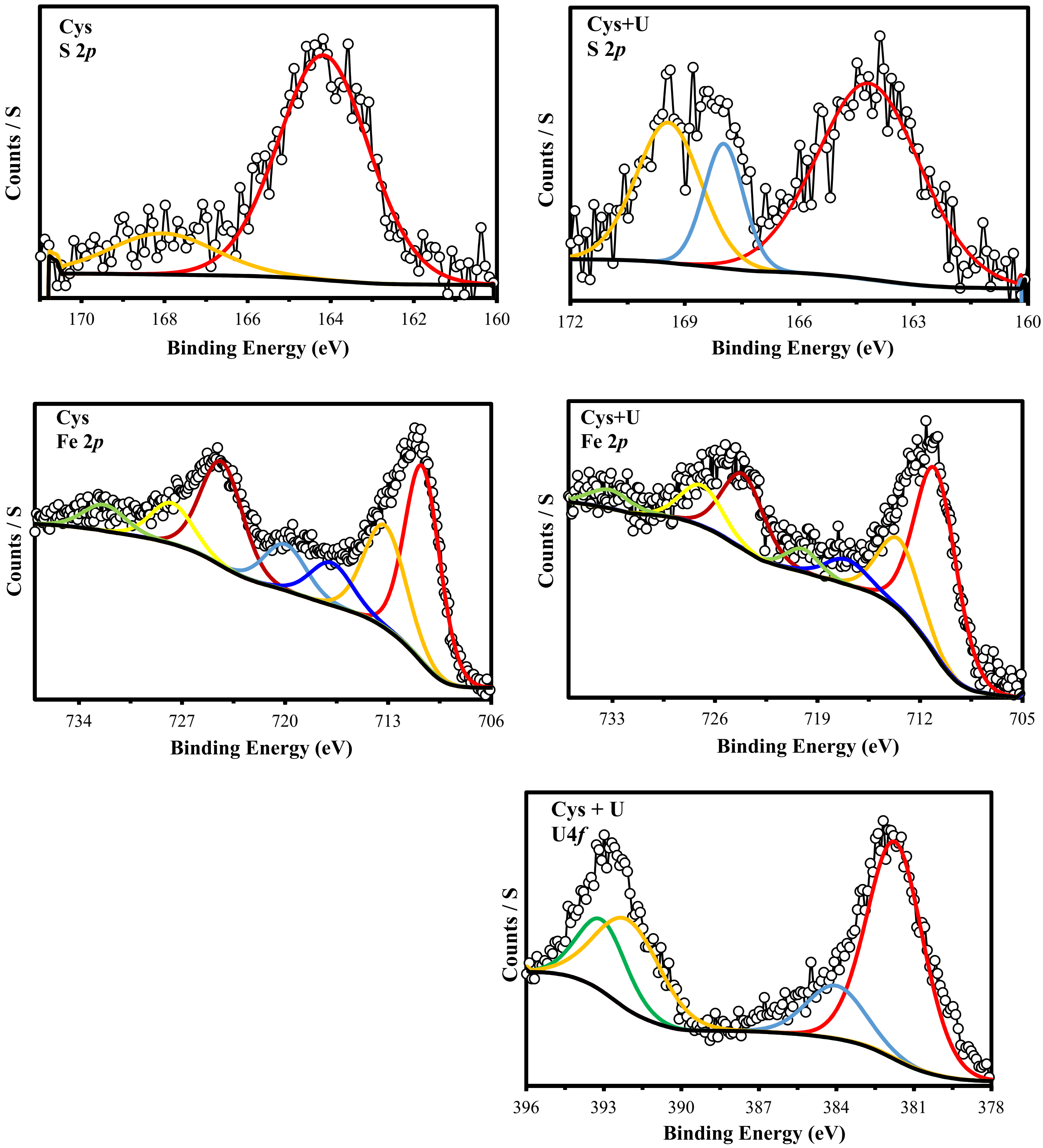

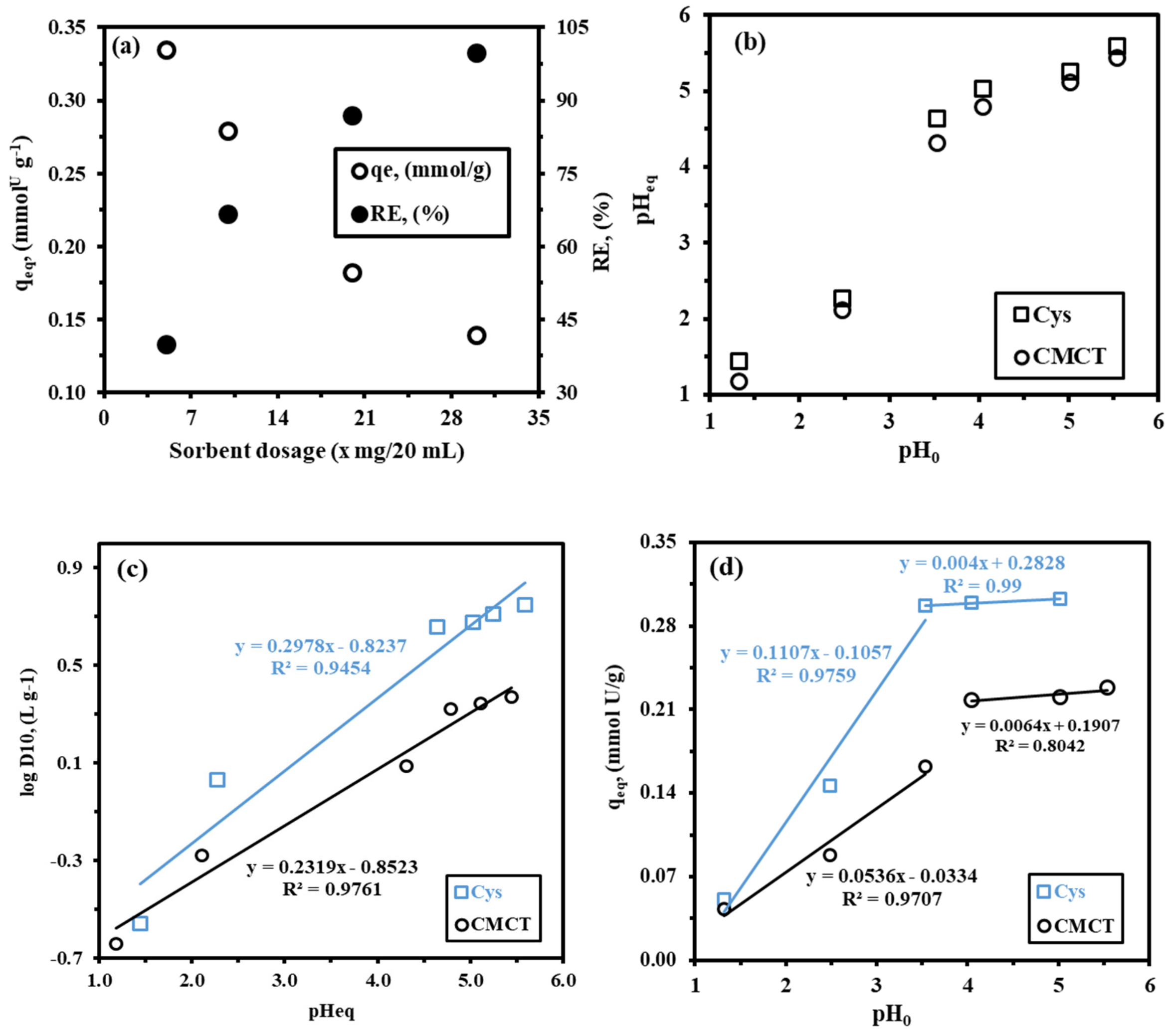

Figure S2. Effect of sorbent dosage (a), Plot of pH0 vs. pH_{eq} (b), $log_{10}D$ plot vs. pH_{eq} (b), and plot of pH_0 vs. q_{eq} (c) for UO_2^{2+} sorption.

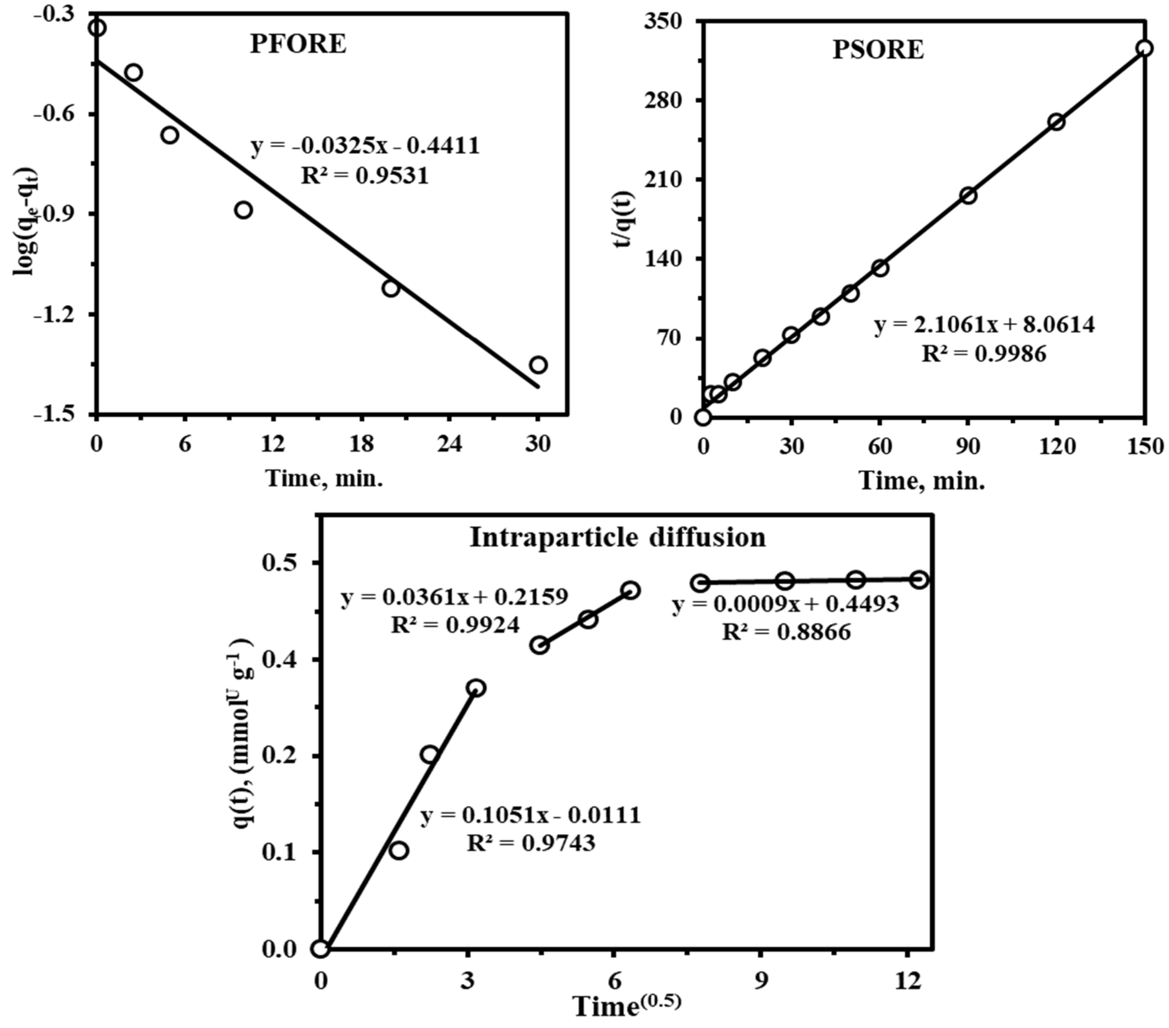

Figure S3. Sorption kinetics for PFORE, PSORE, and Intraparticle diffusion. (C_0: 0.425 mmol U/L, SD: 0.5g/L, time 60 min., room temp. 298 K)

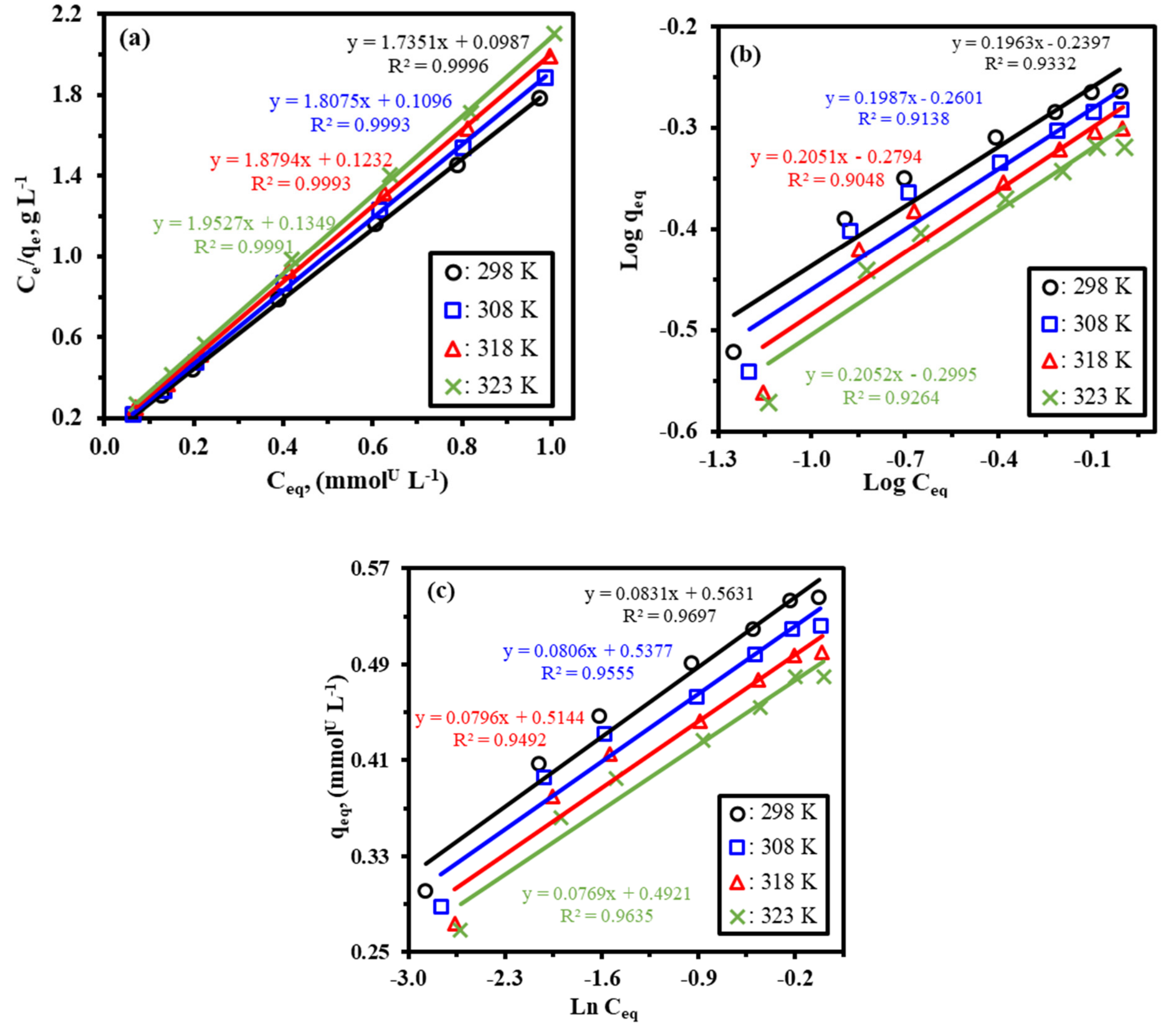

Figure S4. Linearization plots of Langmuir (a), Freundlich (b), and Timken isotherm (c): for UO_2^{2+} sorption. (pH_0: 4.0, SD: 0.5 g L^{-1}, C_0: 0.21–1.26 mmol U/L; T: 298–328 K, Time: 60 min.).

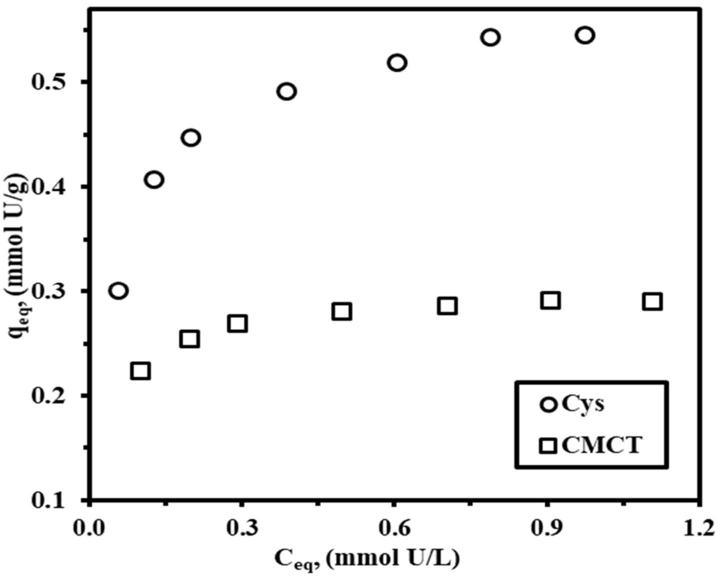

Figure S5. Sorption isotherm using both CMCT and Cys nanocomposites for UO_2^{2+} sorption. (pH_0: 4.0, SD: 0.5 g/L, C_0: 0.21–1.26 mmol U/L, T: 298–328 K, Time: 60 min for Cys and 180 min for CMCT).

References

[1] X. Hu, C. Chen, D. Zhang, Y. Xue, Kinetics, isotherm and chemical speciation analysis of Hg(II) adsorption over oxygen-containing MXene adsorbent, Chemosphere 278 (2021) 130206.
[2] C. Tien, Adsorption calculations and modeling, Butterworth-Heinemann, Boston, 1994.
